# An Alkaline Protease-Digestion of Silkworm Powder Enhances Its Effects Over Healthspan, Autophagy, and Mitochondria Function in a Rotenone-Induced *Drosophila* Model

**DOI:** 10.3389/fnut.2022.808295

**Published:** 2022-06-16

**Authors:** Linh Xuan Mai, Sang-Kug Kang, You-Young Jo, Phuong Nguyen, A-Young Kim, Kee-Young Kim, Nam-Suk Kim, Young Ho Koh

**Affiliations:** ^1^Department of Biomedical Gerontology, Hallym University Graduate School, Chuncheon-si, South Korea; ^2^Industrial Insect and Sericulture Division, National Institute of Agricultural Science, Wanju-gun, South Korea; ^3^Ilsong Institute of Life Science, Hallym University, Seoul, South Korea

**Keywords:** HongJam, protease, mitochondria, autophagy, healthspan, rotenone-induced Parkinson’s disease

## Abstract

**Background:**

Recent studies have reported that steamed and freeze-dried mature silkworms, also known as HongJam, have various health-promoting effects.

**Objective:**

The goal of this study was to elucidate changes in the various health-promoting effects of HongJam, after its digestion with a food-grade protease.

**Materials and Methods:**

We examined whether healthspan-promotion and rotenone-induced loss of motor-control prevention effects were enhanced in *Drosophila* fed with food-grade alkaline protease-digested HongJam compared to those fed with non-digested HongJam. The differences in mitochondrial functions, chemical susceptibilities, and activations of signal transduction pathways between *Drosophila* supplemented with various feed were examined to elucidate the molecular and biochemical basis of healthspan-promotion and locomotor-improvement effects of protease-digested HongJam.

**Results:**

We first found that the healthspan-promotion effect of HongJam digested with a food-grade protease was different depending on the silkworm variety used for its production. Digestion with food-grade protease into White-Jade HongJam (WJ) as prepared from the White-Jade silkworm variety that spins white cocoons did not enhance its functionality. However, compared to Golden-Silk HongJam (GS), a food-grade protease-digested Golden-Silk HongJam (GSD) produced from the Golden-Silk silkworm variety that spins yellow cocoons, it further promoted the healthspan in a *Drosophila* model. By conducting a series of studies to reveal the molecular and biochemical basis for healthspan-promoting effects, we found that GS and GSD similarly enhanced mitochondrial activity, but GSD activated autophagy signaling more than GS. In addition, GSD feed (GSDf)-, GSD supernatant feed (GSDsupf)-, and GSD precipitate feed (GSDprecf)-reared *Drosophila* were also found to have increased resistance to an autophagy inhibitor compared to that of normal feed- or GS feed-reared *Drosophila*. Furthermore, we found that the rotenone-induced loss of motor control prevention effect was superior for GSDsup compared to GS, GSD, or GSDprec. This result may have occurred because GSDsup has more phenolic compounds and antioxidant activities than other samples.

**Conclusion:**

GSDsup contained more digested small peptides and free phytochemicals than other samples due to the digestion of proteins with a food-grade protease. Thus, GSDsup leads to further healthspan-promoting and locomotor-improvement effects than GS, GSD, or GSDprec.

## Introduction

Since silk moths were bred by humans starting 5,000 years ago, they have provided silk fibers for making fabrics and their pupae have been a source of high-quality proteins and fats for humans for a long time (1). In addition, silkworm eggshells, excrements, larval molts, *Bombycis Corpus cum Batryticatus*, and silk moth extracts have been used as traditional Oriental medicines for treating various diseases, such as diabetes, hypertension, fever, stroke, and cerebral infarct (2). Since the 1990s, investigations supporting the scientific bases for various health improvement effects of silkworms and their byproducts have been attempted. As a result, the various health-promoting effects of silkworms and their byproducts recorded in ancient traditional Oriental medicine books for many years have been supported through preclinical and/or clinical studies in recent years. Representative effects include the hypoglycemic effect of freeze-dried 5th instar 3rd-day silkworms (3–5), the sexual function-enhancing effect of male silk moth extracts (6), and the memory enhancement effect of silk Fibroin protein hydrolysates (7, 8). Recently, a processing method has been developed that makes it possible to consume mature silkworms containing enlarged silk glands (9, 10). After the 5th instar on the 3rd-day, silkworms start to develop silk glands. The 5th instar 7th- or 8th-day silkworms, known as mature silkworms, have degenerated internal organs and enlarged silk glands filled with silk fiber proteins (9). Therefore, mature silkworms must have various health improvement effects originating from their diverse functional nutrients (2). In fact, steamed and freeze-dried mature silkworms, also known as HongJam, have been reported to have memory enhancements in mild cognitive impairment rodent models (11, 12), preventing the onset of rotenone-induced loss of motor control (13–15), gastrointestinal protection (16, 17), liver function improvement (18), skin whitening (19), and promotion of the lifespan and healthspan (10, 13, 14).

Since *Drosophila* has an anatomically separated brain, evolutionarily conserved important signal transduction pathways in its genome, short lifespan, and established various research methods for measuring locomotor ability, it has been used as an important animal model for investigating aging and longevities (20, 21). Healthspan was applied for the first time in the study of aging using the *Caenorhabditis elegans* model, and the most relevant to healthy aging of *C. elegans* is mobility (22). Similarly, *Drosophila* also needs to fly or walk to survive, so the most important factor for healthy aging is locomotor ability (13, 14, 23, 24). Therefore, maintenance of voluntary locomotor ability has been used as the most important factor in determining the healthspan of *C. elegans* (22) and *Drosophila* (10, 13, 24). In previous studies, we have shown that *Drosophila* fed with HongJam showed promotion of lifespan and healthspan (10, 13, 14).

Parkinson’s disease (PD) is a progressive degenerative disorder of the central nervous systems caused by genetic defects and/or environmental risk factors in humans. Behavioral symptoms of PD are including slowness of movement, tremor, rigidity, and difficulty with walking. The pathological hallmark of PD is the death of dopaminergic neurons in human brains (25). Among various animal models for PD, *Drosophila* has been used for investigating molecular and cellular mechanisms underlying PD caused by genetic defects or environmental risk factors. For example, null mutations of Parkin, DJ-1, or LRKK2 in *Drosophila* faithfully replicated behavioral symptoms and loss of dopaminergic neurons in brains. In addition, rotenone or paraquat known to cause PD in humans also induced PD in *Drosophila* (26, 27). The molecular etiologies observed in PD patients and animal models included abnormalities in unfolded protein response (UPR), autophagy, mammalian target of rapamycin, and mitochondrial function (28, 29). In previous studies, we have shown that *Drosophila* fed with HongJam showed prevention of the onset of rotenone-induced loss of motor control (10, 13, 15). However, it is not still investigated whether HongJam can enhance UPR, autophagy, Target of rapamycin (Tor), and mitochondrial functions in *Drosophila*.

While we analyzed the nutritional compositions and health enhancement effects between HongJam produced with various pulverization methods, we found that the health enhancement effects of HongJam depended on the sizes of particles and recovery rates (9, 30, 31). Even though smaller HongJam particles gave rise to more effects, the currently available pulverization method for generating approximately 1 μm-sized HongJam particles induced the loss of quite an amount of HongJam particles because of mechanical defects (19).

HongJam consists of approximately 70% crude proteins, 15% crude fatty acids, 3% crude ash, and 2% phytochemicals and vitamins (10, 19, 30, 32). Recently, the Fibroin in silk fibers could reportedly be hydrolyzed by food-grade proteolytic enzymes (33). The FoodPro^®^ alkaline protease (FP^®^ AP) was efficiently used to hydrolyze the silk fiber Fibroin. Thus, we investigated whether digesting HongJam with FP^®^ AP can enhance healthspan promotion and rotenone-induced loss of motor control prevention effects of HongJam.

## Materials and Methods

### Rearing Mature Silkworms and Producing HongJam

White-Jade and Golden-Silk varieties of *Bombyx mori* were raised on mulberry leaves at the campus of the National Institute of Agricultural Science (NIAS), Wanju-gun, Jeolla-buk do, South Korea. HongJam, also known as steamed and freeze-dried mature silkworms, was produced as previously published (9, 30). Voucher specimens of White-Jade HongJam (WJ) and Golden-silk HongJam (GS) were deposited in the *Bombyx mori* Quality Maintenance and Storage Laboratory, Division of Industrial Insect and Sericulture, NIAS, Wanju-gun, Jeollabuk-do, South Korea.

### Digestion of HongJam With a Food-Grade Protease

A previously published protocol was used (34). In brief, to prepare enzymatically digested WJ (WJD) and GS (GSD), 150 g of WJ or GS was mixed with 75 ml of FP^®^ AP (DuPont Industrial Biosciences, Brabrand, Denmark). After dH_2_O was added to the mixtures to make 1 L of solution, an enzyme digestion was performed in a rotary stirrer at 55°C for 24 h. The FP^®^ AP was deactivated by boiling at 90°C for 10 min. WJD and GSD were harvested and separated with supernatants and precipitates by centrifuging at 13,000 rpm for 10 min. The supernatants and precipitates were freeze-dried, and the weight ratio of dried supernatants to precipitates was 7:3.

### Size Exclusion Chromatography of Various Samples

To determine the biophysical characteristics of samples, size exclusion chromatography was performed using an AKTA fast protein liquid chromatography system (AKTA FPLC, GE Healthcare, Chicago, IL, United States) equipped with a Superose™ 6 10/300 column (GE Healthcare) equilibrated with 0.1 M phosphate buffer (pH 7.2, 150 mM NaCl). A gel filtration standard markers kit (protein molecular weight 12.4∼200.0 kDa, Merck KGaA, Darmstadt, Germany) was used to calibrate a Superose™ 6 10/300 column. The void volume (Vo) of the Superose™ 6 10/300 column was 7.5 ml. The ratios of elution volume/Vo of 200, 150, 66, 29, and 12.4 kDa protein standards were 1.988, 2.095, 2.174, 2.399, and 2.537, respectively. Radio immuno-precipitation assay (RIPA) buffer (10 mM Tris-HCl, pH 8.0, 1 mM EDTA, 0.5 mM EGTA, 1.0% NP-40, 1.0% sodium deoxycholate, 0.1% SDS, 140 mM NaCl, Merck) containing Halt™ protease inhibitor cocktail (ThermoFisher Scientific, Waltham, MA, United States) was used to extract proteins from samples. 0.3 ml (5.0 mg/ml) of samples were injected into AKTA FPLC and then separated for 70 min with a flow rate of 1.0 ml/min. UV absorbance changes at 215, 254, and 280 nm were monitored to reveal amounts of digested peptides, other bio-molecules with ring structures, and proteins, respectively, in samples.

### *Drosophila* Lifespan and Healthspan Assays

The Canton-S strain of *Drosophila melanogaster* obtained from the Bloomington *Drosophila* Stock Center (Indiana University, Bloomington, IN, United States) was used to test whether the lifespan and healthspan of *Drosophila* supplemented with WJ, WJD, GS, or GSD were altered. *Drosophila* was raised with normal feed [Nf (1.0 L dH_2_O, 7.7 g agar, 62.4 g dried yeast, 40.8 g corn starch, 84.0 g glucose, 13.0 ml molasses, and 12.5 ml mold inhibitor)] or HongJam feeds containing 6.24 g of WJ, WJD, GS, or GSD in Nf. The life expectancy and healthspans were investigated as previously published (10, 13, 24). The healthspan in *Drosophila* was defined as the period with voluntary movement ability in previous reports.

To examine the effects of GSD supernatant (GSDsup) and GSD precipitate (GSDpepc) on *Drosophila* lifespans and healthspans, 6.24 g of GSDsup or GSDprec was mixed with Nf to make GSDsup feed (GSDsupf) or GSDprec feed (GSDprecf). The *Drosophila* were raised with Nf, GS feed (GSf), GSD feed (GSDf), GSDsupf, or GSDprecf, and the numbers of live flies and active flies were counted as previously published (10, 13, 24).

### Assays for Activities of Mitochondrial Complexes I∼IV

The activities of mitochondrial complexes (MitoCom) I∼IV in 5-, 10-, 15-, or 20-day-old *Drosophila* reared with Nf, GSf, or GSDf were measured as previously published (11, 12). Ten *Drosophila* adults were ground in mitochondria lysis buffer (0.25 M sucrose, 5 mM Tris-HCl, 2 mM EGTA, and 1% BSA, pH 7.4), and then the debris was removed by three layers of medical gauze (Dae Han Medical Supply Co. LTD, Chuncheon-si, South Korea). Filtered lysates were centrifuged at 150× *g* for 5 min at 4°C and the supernatants were collected and then centrifuged at 900× *g* for 10 min at 4°C to collect the mitochondrial pellets. The pellets were resolved with 200 μl of lysis buffer and then divided into two tubes: one tube containing 155 μl for MitoCom I∼III and the other tube containing 45 μl of a mitochondrial sample with 5 μl of 10 mM n-D-β-D maltoside for MitoCom IV. All activities of MitoCom I∼IV were normalized by using the activities of *Drosophila* reared with Nf.

### Chemical Susceptibility Assay for *Drosophila*

After treatment with various signal transduction pathway modulators, the changes in the survival rates of *Drosophila* reared with diverse feeds were investigated to elucidate the signaling altered by GS or GSD. Dithiothreitol [DTT, an endoplasmic reticulum (ER) stress inducer, Merck], fipronil [a gamma-aminobutyric acid A-type receptor (GABA_*A*_-R) channel blocker, Tokyo Chemical Industry], H_2_O_2_ (an oxidative stress inducer, Merck), LiCl (an autophagy inducer, DaeJung), and 3-methyladenine (3-MA, an autophagy inhibitor, Merck) were used to modulate various signal transduction pathways. *Drosophila* were reared with various feeds containing GS, GSD, GSDsup, or GSDprep, and then one hundred adults (50 females and 50 males) were exposed to various signal transduction modulators mixed with 1.5% agar with 0.5 M sucrose and incubated at 28.0 ± 1.0°C. The numbers of live flies were counted every 3 days, and then those live flies were transferred to new tubes containing fresh feed with modulators.

### Real-Time Quantitative PCR Protocol

The expression changes of 36 genes related to the unfolded protein response (UPR), autophagy, and the target of rapamycin (Tor)/AKT/phosphatidylinositol 3-kinase (PI3K) were investigated by performing real-time quantitative PCR (RT–qPCR). In addition, the expression changes of six gustatory receptors (Gr), Gr64af, were investigated. The heads and bodies of 7- or 15-day-old adult flies were collected to extract the total RNA using TRIzol reagents according to the manufacturer’s protocol. After the quality of the total RNA was determined using the A260/A280 and 28S rRNA/18S rRNA ratios, DNase I (Promega, Madison, WI, United States) was used to remove genomic DNA contaminants, and then cDNA was synthesized using Superscript IV (Thermo Fisher Scientific). The DNA sequences of the oligomers and PCR conditions are listed in Supplementary Table 1. RT-qPCRs using SYBR green master mix (Thermo Fisher Scientific) were performed using an ARIA MX RT-PCR machine and software (Agilent, Santa Clara, CA, United States). Three biological replications were performed for each analysis. The previously published 2^–ΔΔCT^ method was used to quantify the relative expression levels of the genes.

### Gene-to-Gene Interaction Analysis

The STRING database (DB)^1^ (35) was used to perform a gene-to-gene interaction analysis of differentially expressed genes (DEGs) in the heads and bodies of 7- or 15-day-old *Drosophila*.

### Survival Analysis for Rotenone-Induced *Drosophila* Model

To investigate the effects of GS, GSD, GSDsup, or GSDprec on the onset and progression of rotenone-induced loss of motor control in a *Drosophila* model, Nf-, GSf-, GSDf-, GSDsupf-, or GSDprecf-reared *Drosophila* were treated with 0.2 M rotenone in 0.1% dimethyl sulfoxide (DMSO), 1.5% agar, and 0.5 M sucrose (Dae-Jung). One hundred age-matched adult *Drosophila* (50 females and 50 males) were collected and then exposed to 0.2 M rotenone. For the controls, Nf-, GSf-, GSDf-, GSDsupf-, or GSDprecf-reared *Drosophila* were exposed to only 0.1% DMSO in 1.5% agar and 0.5 M sucrose. The number of live *Drosophila* was counted every 3 days. Kaplan-Meier survival analyses were performed as described below.

### Determining the Phytochemicals in GS, GSD, GSDsup, and GSDprec

To quantify the amounts of flavonoids or polyphenolic compounds in various samples, the samples were mixed with 80% methanol (MeOH, vol/vol) and then shaken for 90 min at 150 rpm using a rotary shaker (IS971R, Jeio Tech, DaeJeon, South Korea). Mixed solutions were centrifuged to obtain their supernatants. Following filtration with a syringe filter (0.2 μm pore size, Sartorius AG, Gottingen, Germany) to remove small debris, the amounts of total phenolic compounds and flavonoids were measured as previously published (11, 14, 36). The amounts of phytochemicals in GSD, GSDsup, and GSDprec were normalized by using those of GS.

### Antioxidant Activity Assays

The antioxidant activities of GS, GSD, GSDsup, and GSDprep were examined by performing a 1,1-di-phenyl-2-picryl-hydrazyl (DPPH) assay and a ferric-reducing ability of plasma (FRAP) assay as previously published (11, 14, 36). The antioxidant activities in GSD, GSDsup, and GSDprec were normalized by using those of GS.

### Statistical Analysis

For comparing mitochondrial activities, amounts of phytochemicals, and antioxidant activities, one-way analysis of variance (ANOVA) and Tukey’s honestly significant difference (HSD) *post-hoc* analysis were performed using Microsoft Excel for Windows 10 (Microsoft, Redmond, WA, United States) as previously published (11, 12). All results were presented as the mean ± the standard error of the mean.

Kaplan-Meier survival estimations were performed to draw the survival or locomotor ability curves of *Drosophila* fed with different feeds or treated with various chemicals. In addition, Cox proportional hazard regression analyses and the log-rank tests were performed to obtain hazard ratios (HRs) and 95% confidential intervals (CIs), and *p*-values, respectively. Kaplan-Meier survival estimations, the log-rank tests, and Cox proportional hazard regression analyses were performed using the R program (version 4.0.3; The R Foundation) as previously published (12, 37).

## Results

### Digestion of HongJam With a Food-Grade Protease Altered Its Biophysical Characteristics

The size exclusion chromatograms of RIPA extracts of GS, GSD, GSDsup, or GSDprec revealed unique biophysical characteristics of samples (Supplementary Figure 1). The chromatogram of a RIPA extract of GS had two separate parts (Supplementary Figure 1A). The size of the 1st part was smaller than that of the 2nd part. In contrast, the chromatogram of a RIPA extract of GSD showed the tiny 1st part and the large 2nd part (Supplementary Figure 1B). In addition, the chromatograms of a RIPA extract of GSDsup showed a unique pattern. The narrow and pointed 1st part appeared close to the large 2nd part (Supplementary Figure 1C). This unique chromatogram of GSDsup suggested that digestion of HongJam with a food-grade protease might cause digestion of high molecular weight proteins to low molecular weight peptides. The chromatogram of a RIPA extract of GSDprec differed from others. The 1st part was larger than those of other samples (Supplementary Figure 1D). This result indicated that peptidoglycans, major components of larval cuticles, might be digested by a food-grade protease, and decomposed high molecular weight molecules were released from GSDprec while extracting with a RIPA buffer. The unique chromatograms of samples might ensure the quality of the samples.

### Further Extension of Healthspan in *Drosophila* Supplemented With GSD

**FIGURE 1:**
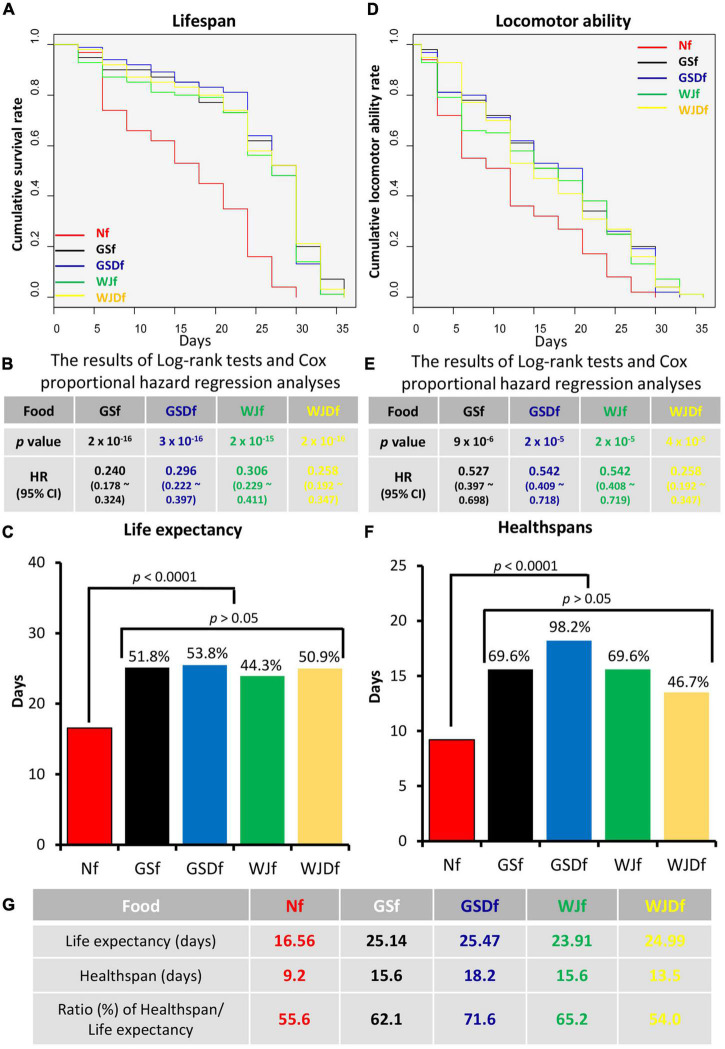
The life expectancy and healthspan of *Drosophila* reared with various feeds. **(A)** The lifespans of *Drosophila* reared with GS feed (GSf), GSD feed (GSDf), WJ feed (WJf), or WJD feed (WJDf) were extended compared with that of Normal feed (Nf)-reared *Drosophila.* **(B)** When the life expectancy of *Drosophila* reared with GSf, GSDf, WJf, or WJDf was compared with that of Nf-reared *Drosophila*, the hazard ratios (HRs) were significantly reduced (*p* < 0.005). **(C)** Compared to Nf-reared *Drosophila*, the average lifespans of GSf-, GSDf-, WJf-, or WJDf-reared *Drosophila* were increased. **(D)** Compared with Nf-reared *Drosophila*, the voluntary locomotor abilities of GSf-, GSDf-, WJf-, or WJDf-reared *Drosophila* were significantly increased. The period of *Drosophila* retained voluntary locomotor ability was defined as the healthspan. **(E)** The HRs of GSf-, GSDf-, WJf-, and WJDf-reared *Drosophila* were significantly reduced (*p* < 0.005). **(F)** The healthspans of GSf-, GSDf-, WJf-, or WJDf-reared *Drosophila* were significantly increased. **(G)** The ratios of life expectancies and healthspans were higher in GSDf-, GSf-, and WJf- but lower in WJDf-reared *Drosophila* than in Nf-reared *Drosophila*.

The lifespans and healthspans of *Drosophila* reared with WJf, WJDf, GSf, or GSDf were compared with those of Nf-reared *Drosophila* (Figure 1). All the *Drosophila* reared with WJf, WJDf, GSf, and GSDf showed a statistically significant decrease in HRs (Figures 1A,B). Compared with Nf-reared *Drosophila*, the HRs of GSf-, GSDf-, WJf-, or WJDf-reared *Drosophila* were 0.240, 0.296, 0.306, or 0.258, respectively. However, there was no significant difference between the various HongJam feed-reared groups. When the life expectancy was converted into the average lifespan, the average lifespan of Nf-reared *Drosophila* was 16.56 days, while the average lifespans of GSf-, GSDf-, WJf-, or WJDf-reared *Drosophila* were 25.14 days (51.8% up), 25.47 days (53.8% up), 44.4% to 23.91 days (44.4% up), or 24.99 days (50.9% up), respectively (Figure 1C).

When the locomotion ability was examined to calculate the healthspan of *Drosophila*, we found that the locomotion ability of GSf-, GSDf-, WJf-, or WJDf-reared *Drosophila* was significantly increased compared to that of Nf-reared *Drosophila*. The HRs of GSf-, GSDf-, WJf-, and WJDf-reared *Drosophila* were significantly decreased to 0.527, 0.542, 0.542, and 0.553, respectively, compared to those of Nf-reared *Drosophila* (*p* < 0.005, Figures 1D,E). In previous studies, the healthspan of *Drosophila* was defined as the point at which 50% of the individuals had locomotor ability (10, 13, 14). Although the healthspan of Nf-reared *Drosophila* was only 11.9 days, those of GSf-, GSDf-, WJf-, or WJDf-reared *Drosophila* were extended to 15.6 days (69.6% up), 18.2 days (98.2% up), 15.6 days (69.6% up), or 13.5 days (46.7% up), respectively (Figure 1F).

As the ratio of healthspans to life expectancies increased, the period of suffering from disease decreased (22, 38), so this ratio is one of the important factors to confirm health promotion. The ratio of the healthspan to the average lifespan of Nf-reared *Drosophila* was 55.6%, whereas that of GSf-, GSDf-, or WJf-cultured *Drosophila* increased up to 62.1, 71.6, or 65.2%, respectively. However, that of WJDf-reared *Drosophila* decreased to 54.0% (Figure 1G). The ratio of healthspan/life expectancy was deteriorated in WJD compared to WJ. These results showed that GS and GSD were more effective at increasing the healthspan than WJ and WJD. Thus, further research was conducted using GS and GSD.

### Enhancement of MitoCom I∼IV Activities in *Drosophila* by GS and GSD

In previous studies, we showed that *Drosophila* and mice fed GS showed enhanced mitochondrial function (11, 12). Thus, we investigated whether the activities of MitoComs I∼IV in *Drosophila* reared with GSf or GSDf were different from those of Nf-reared *Drosophila*. The MitoCom I activities were highest in GSf-reared *Drosophila* from Day 5 to Day 15 but in GSDf-reared *Drosophila* on Day 20 (Supplementary Figure 2A). Similarly, the MitoCom II activities were highest in GSf-reared *Drosophila* from Day 5 to Day 15. On Day 20, GSf- and GSDf-reared *Drosophila* had significantly higher MitoCom II activities than Nf-reared *Drosophila* (Supplementary Figure 2B). The activity of MitoCom III was highest in GSf-reared *Drosophila* on Day 5 and Day 15, and GSDf-reared *Drosophila* had the highest activity on Day 10. On Day 20, both GSf- and GSDf-reared *Drosophila* showed higher activity than Nf-reared *Drosophila* (Supplementary Figure 2C). The activity of MitoCom IV in GSf- and GSDf-reared *Drosophila* was significantly higher on Day 10 than that in Nf-reared *Drosophila*. On the other days, GSf-reared *Drosophila* had the highest MitoCom IV activity (Supplementary Figure 2D).

These results suggested that the enhanced activities of MitoCom I∼IV might be in part the molecular basis for the extended life expectancy and healthspans in GSf- and GSDf-reared *Drosophila.*

### GS and GSD Spatiotemporally Differentially Regulate Autophagy, Tor, and UPR Signaling

In previous studies, we showed that the onset and progression of rotenone-induced loss of motor control in *Drosophila* was prevented by GS. UPR, autophagy, mTor signaling, and mitochondrial function were shown to be involved with longevities and loss of motor control (28, 29). Thus, we investigated the DEGs in the heads and bodies of 7- or 15-day-old *Drosophila* reared with Nf, GSf, or GSDf.

**FIGURE 2:**
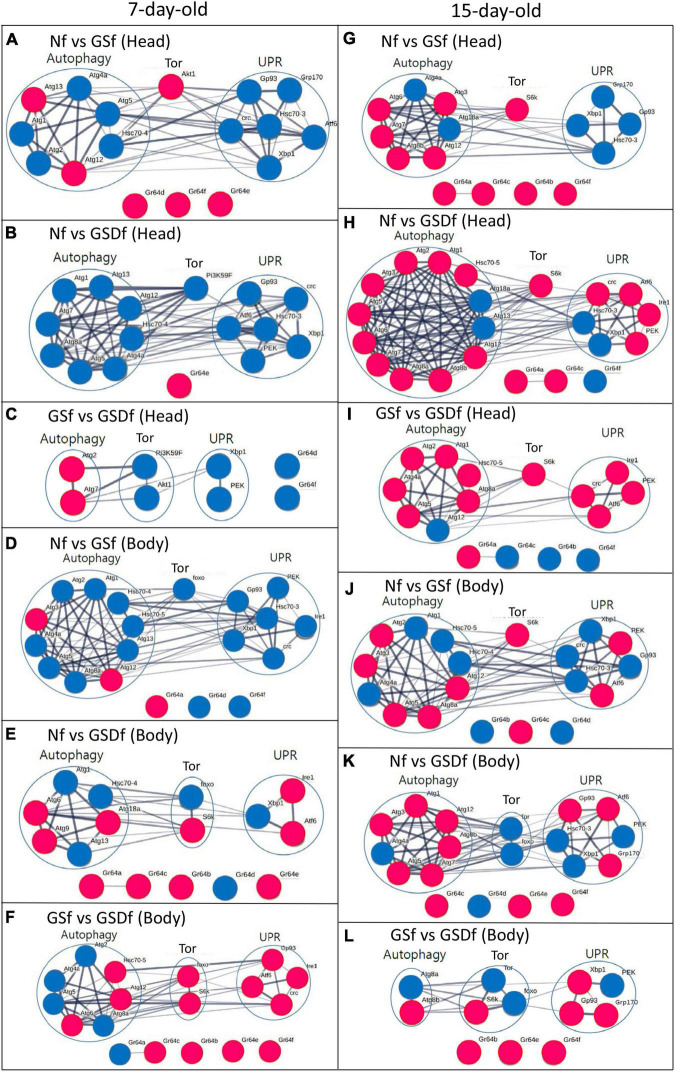
Results of gene-to-gene interaction analysis of differentially expressed genes (DEGs) in autophagy-, Unfolded protein response (UPR)-, and Tor-signaling in the heads and bodies of 7- or 15-day-old Nf-, GSf-, and GSDf-reared *Drosophila*. Red circles indicate upregulated DEGs, while blue circles indicate downregulated DEGs. DEGs in the heads of 7-day-old GSf- **(A)** or GSDf-reared *Drosophila* **(B)** compared with those of Nf-reared *Drosophila*. DEGs in the heads of 7-day-old GSDf-reared *Drosophila* compared with those of GSf-reared *Drosophila* **(C)**. DEGs in the body of 7-day-old GSf- **(D)** or GSDf-reared *Drosophila* **(E)** compared with that of Nf-reared *Drosophila*. DEGs in the heads of 7-day-old GSDf-reared *Drosophila* compared with those of GSf-reared *Drosophila* **(F)**. DEGs in the heads of 15-day-old GSf- **(G)** or GSDf-reared *Drosophila* **(H)** compared with that of Nf-reared *Drosophila*. DEGs in the heads of 15-day-old GSDf-reared *Drosophila* compared with those of GSf-reared *Drosophila* **(I)**. DEGs in the bodies of 15-day-old GSf- **(J)** or GSDf-reared *Drosophila* **(K)** compared with those of Nf-reared *Drosophila*. DEGs in the bodies of 15-day-old GSDf-reared *Drosophila* compared with that of GSf-reared *Drosophila* **(L)**.

In comparison with the expression of genes in the heads of 7-day-old Nf-reared *Drosophila*, the expression of 11 or 6 genes in the heads of 7-day-old GSf-reared *Drosophila* was significantly reduced or increased, respectively (Supplementary Table 2). Five out of 7 DEGs (Atg1, Atg2, Atg4a, Atg5, and Hsc70-4) associated with autophagy signaling or 6 DEGs (Atf6, Gp93, Grp170, crc, Hsc70-3, and Xbp 1) in UPR signaling were downregulated, while the expression of AKT1 in Tor signaling and three Grs (Gr64d, Gr64f, and Gr64e) were upregulated compared with those of Nf-reared *Drosophila* (Figure 2A). Similarly, compared with the body of 7-day-old Nf-reared *Drosophila*, the expression of 15 genes decreased and only one increased in the body of 7-day-old GSDf-reared *Drosophila* (Supplementary Table 2). Eight DEGs in autophagy signaling (Atg1, Atg4, Atg5, Atg7, Atg8a, Atg12, Atg13, and Hsc70-4), one DEG in Tor signaling (Pi3K59F), and 6 DEGs in UPR signaling (Atf6, crc, Gp93, Hsc70-3, PEK, and Xbp1) were all downregulated, and only Gr64e was upregulated (Figure 2B). When comparing GSf- and GSDf-reared *Drosophila*, two DEGs in autophagy signaling (Atg2 and Atg7) were upregulated, and 2 DEGs in Tor signaling (Pi3K59F and Akt1), UPR signaling (Xbp1 and PEK) and Grs (Gr64d and Gr64f) were downregulated (Figure 2C). More DEGs were found in the bodies of 7-day-old *Drosophila* reared with GSf or GSDf than in Nf-reared *Drosophila* (Supplementary Table 2 and Figures 2D–F). Compared with Nf-reared *Drosophila*, 19 down- and three upregulated DEGs were observed in GSf-reared *Drosophila*. Eight out of 10 DEGs in autophagy signaling (Atg1, Atg2, Atg4a, Atg5, Atg8a, Atg13, Hsc70-4, and Hsc70-5), one DEG in Tor signaling (foxo), all six DEGs in UPR signaling (crc, Gp93, Hsc70-3, Ire1, PEK, and Xbp1), and two out of three Grs (Gr64d and Gr64f) were downregulated (Figure 2D). By contrast, more DEGs were upregulated in GSDf-reared *Drosophila* than in Nf-reared *Drosophila*. Three out of six DEGs in autophagy signaling (Atg1, Atg13, and Hsc70-4), one out of two Tor signaling (foxo), one out of three DEGs in UPR signaling (Xbp1), and one out of five DEGs in Grs (Gr64d) were downregulated (Figure 2E). When GSf- and GSDf-reared *Drosophila* were compared, three out of seven DEGs in autophagy signaling (Atg6, Atg12, and Hsc70-5), two DEGs in Tor signaling (foxo and S6k), all four UPR signaling (Atf6, crc, Gp93, and Ire1), and four out of five DEGs in Grs (G64b, Gr64c, Gr64e, Gr6f) were upregulated. Except for the Grs, the DEGs showed very strong intra- and inter-signaling interactions (Figures 2A–F).

The expression patterns of DEGs in the heads and bodies of 15-day-old *Drosophila* reared with GSf or GSDf compared to those of Nf-reared *Drosophila* were different from those of 7-day-old *Drosophila* (Supplementary Table 3 and Figures 2G–L). Compared with the heads of 15-day-old Nf-reared *Drosophila*, five out of seven DEGs in autophagy signaling (Atg3, Atg6, Atg7, Atg8b, and Atg12), one DEG in Tor signaling (S6k), and all four Grs (Gr64a, Gr64b, Gr64c, and Gr64f) were upregulated, while all four DEGs in UPR signaling (Hsc70-3, Grp170, Gp93, and Xbp1) were downregulated (Figure 2G). More DEGs were revealed when GSDf-reared *Drosophila* was compared with Nf-reared *Drosophila* (Figure 2H). Ten out of 12 DEGs in autophagy signaling (Atg1, Atg2, Atg3, Atg5, Atg6, Atg7, Atg8a, Atg8b, and Atg12), one DEG in Tor signaling (S6k), and four out of six DEGs in UPR signaling (Atf6, crc, Ire1, and PEK) were upregulated in the heads of 15-day-old GSDf-reared *Drosophila*. When GSf- and GSDf-reared *Drosophila* were compared, six out of seven DEGs in autophagy signaling (Atg1, Atg2, Atg4a, Atg5, Atg8a, and Hsc70-5), one DEG in Tor signaling (S6k), all four DEGs in UPR signaling (Atf6, crc, Ire1, and PEK), and one out of four DEGs (Gr64a) in Grs were upregulated (Figure 2I). When the expression of genes in the body of 15-day-old GSf-reared *Drosophila* was compared with that of Nf-reared *Drosophila*, five out of nine DEGs in autophagy signaling (Atg2, Atg3, Atg5, Atg8a, and Atg12), one DEG in Tor signaling (S6k), two out of six DEGs in UPR signaling (Atf6 and PEK), and one out of three DEGs in Grs (Gr64c) were upregulated (Figure 2J). In the case of GSDf-reared *Drosophila*, one out of seven DEGs in autophagy signaling (Atg4a), two DEGs in Tor signaling (Tor and foxo), three out of six DEGs in UPR signaling (Hsc70-3, PEK, and Xbp1), and one out of four DEGs in Grs (Gr64d) were downregulated (Figure 2K). In addition, when GSf- and GSDf-reared *Drosophila* were compared, one out of two DEGs in autophagy signaling (Atg8b), one out of three DEGs in Tor signaling (S6k), three out of four DEGs in UPR signaling (Gp93, Grp170, and Xbp1), and all three DEGs in Grs (Gr64c, Gr64e, and Gr64f) were upregulated (Figure 2L). Except for the DEGs in the Grs, there were strong intra- and inter-signaling interactions among DEGs (Figures 2G–L).

Taken together, the DEG analysis results showed that GS and GSD had different spatiotemporal effects on the heads and bodies of 7- and 15-day-old *Drosophila*. Since GSDf-reared *Drosophila* had more up-regulated DEGs in autophagy and UPR signaling than GSf-reared *Drosophila*, the functional nutrients in enzyme-digested GSD might be more easily absorbed than those in GS, resulting in enhancing autophagy and UPR signaling in 15-day-old *Drosophila*.

### GS and GSD Alter the Susceptibility of *Drosophila* to Modulators of Various Signal Transduction Pathways

*Drosophila* reared with GSf and GSDf showed significantly extended life expectancies and healthspans (Figure 1). To investigate which signal transduction pathways were altered, we investigated the alteration of sensitivities in *Drosophila* to the modulators of various signal transduction pathways (Table 1).

**TABLE 1:** Altered susceptibility of GS feed (GSf)-, GSD feed (GSDf)-, GSD supernatant feed (GSDsupf)-, and GSD precipitate feed (GSDprecf)-reared *Drosophila* to various signal transduction pathway modulators.

Chemicals		DTT^3^		Fipronil		H_2_O_2_		LiCl	3-MA^4^
		ER stress inducer		GABA_*A*_-R blocker		Oxidative stressor		Autophagy inducer inhibitor	
Concentration		50 mM	0.1 M	10 nM	50 nM	0.3%	1.5%	50 mM	20 mM
GS	HR^1^	1.2510	0.7160 ↓	0.6959 ↓	0.7080 ↓	4.274 ↑	1.571 ↑	0.9383	0.8055
	*p*-value	>0.05	<0.05	<0.05	<0.05	<0.005	<0.005	>0.05	>0.05
	95% CI^2^	0.9476∼1.1651	0.5418∼0.9426	0.5234∼0.9252	0.5304∼0.945	3.099∼5.893	1.183∼2.085	0.5794∼1.013	0.6039∼1.0744
GSD	HR^1^	0.4767 ↓	0.5022 ↓	0.9669	0.8669	1.923 ↑	2.0986 ↑	0.7662	0.1705 ↓
	*p*-value	<0.005	<0.005	>0.05	>0.05	<0.005	<0.005	>0.05	<0.005
	95% CI^2^	0.3518∼0.646	0.3783∼0.6667	0.7301∼1.280	0.6562∼1.145	1.438∼2.571	1.579∼2.790	0.5794∼1.013	0.1218∼0.2387
GSDsup	HR^1^	1.6024 ↑	1.348 ↑	0.7029 ↓	0.6414 ↓	2.897 ↑	0.9339	0.957	0.1761 ↓
	*p*-value	<0.01	<0.05	<0.05	<0.005	<0.01	>0.05	>0.05	<0.005
	95% CI^2^	1.209∼2.123	1.018∼1.784	0.5295∼0.9329	0.4806∼0.8559	1.097∼1.931	0.7099∼1.244	0.7383∼1.2892	0.1254∼0.2473
GSDprec	HR^1^	0.8329	1.323 ↑	0.9445	1.447 ↑	1.454 ↑	1.9732 ↑	0.6511 ↓	0.1729 ↓
	*p*-value	>0.05	<0.05	>0.05	>0.01	<0.005	<0.005	<0.005	<0.005
	95% CI^2^	0.625∼1.110	1.000∼1.750	0.7153∼1.2482	1.0938∼1.9143	2.141∼3.919	1.488∼2.616	0.4911∼0.8631	0.1230∼0.2431

*↑, significantly increased.*

*↓, significantly decreased.*

*^1^HR, Hazard ratio; ^2^95% CI, 95% confidential interval; ^3^DTT, Dithiothreitol; ^4^3-MA, 3-methyladenine.*

After the *Drosophila* were treated with the ER stress inducer DTT at 50 and 100 mM in GSf-, GSDf-, GSDsupf-, and GSDprecf-reared *Drosophila*, the survival rate was compared to that of Nf-reared *Drosophila* (Table 1). When GSf-reared *Drosophila* were exposed to DTT, HR (0.7160, *p* < 0.05) was significantly reduced only when exposed to 100 mM DTT. The HRs of GSDf-reared *Drosophila* exposed to 50 mM (0.4767, *p* < 0.005) or 100 mM DTT (0.5022, *p* < 0.005) were significantly reduced. However, GSDsupf-reared *Drosophila* exposed to 50 mM (1.6024, *p* < 0.005) and 100 mM DTT (1.348, *p* < 0.05) showed significantly increased HRs. In addition, GSDprecf-reared *Drosophila* exposed to 100 mM DTT only showed a significantly increased HR (1.323, *p* < 0.05).

*Drosophila* reared with various feeds showed altered HRs when exposed to 10 nM or 50 nM of the GABA_*A*_R blocker fipronil (Table 1). The HRs of GSf-reared *Drosophila* exposed to 10 nM (0.6959, *p* < 0.05) or 50 nM fipronil (0.7080, *p* < 0.05) were significantly reduced. Although the HRs of GSDf-reared *Drosophila* exposed to fipronil did not show any difference from those of Nf-reared *Drosophila*, GSDsupf-reared *Drosophila* exposed to 10 nm (0.7029, *p* < 0.05) or 50 nm fipronil (0.6414, *p* < 0.005) showed significantly reduced HRs. By contrast, GSDprecf-reared *Drosophila* showed a significantly increased HR when exposed to 50 nm fipronil (1.447, *p* < 0.01).

For the oxidative stressor H_2_O_2_, the HRs of 0.3% (4.274, *p* < 0.005) or 1.5% H_2_O_2_ (1.571, *p* < 0.05)-exposed GSf-reared *Drosophila* were significantly increased. Similarly, the HRs of 0.3% (1.454, *p* < 0.005) or 1.5% H_2_O_2_ (1.9732, *p* < 0.005) were significantly increased in GSDf-reared *Drosophila*. However, GSDsupf-reared *Drosophila* only showed a significantly increased HR when exposed to 0.3% H_2_O_2_ (2.897, *p* < 0.01).

When exposed to the autophagy inducer 50 mM LiCl, the HR (0.6511, *p* < 0.005) of GSDprecf-reared *Drosophila* was significantly reduced. By contrast, when exposed to the autophagy inhibitor 20 mM 3-MA, the HRs of GSDf- (0.1705, *p* < 0.005), GSDsupf- (0.1761, *p* < 0.005), and GSDprecf (0.1729, *p* < 0.005) were significantly reduced.

Taken together, these results suggested that the signal transduction pathways affected by GS and GSD might be different. Nevertheless, consistent with the DEG analysis results, GSD-, GSDsup-, and GSDprec-reared *Drosophila* revealed enhanced resistance to an autophagy inhibitor.

### Preventing the Onset of Rotenone-Induced Loss of Motor Control in GSf-, GSDf-, GSDsupf-, and GSDprecf-Reared *Drosophila*

**FIGURE 3:**
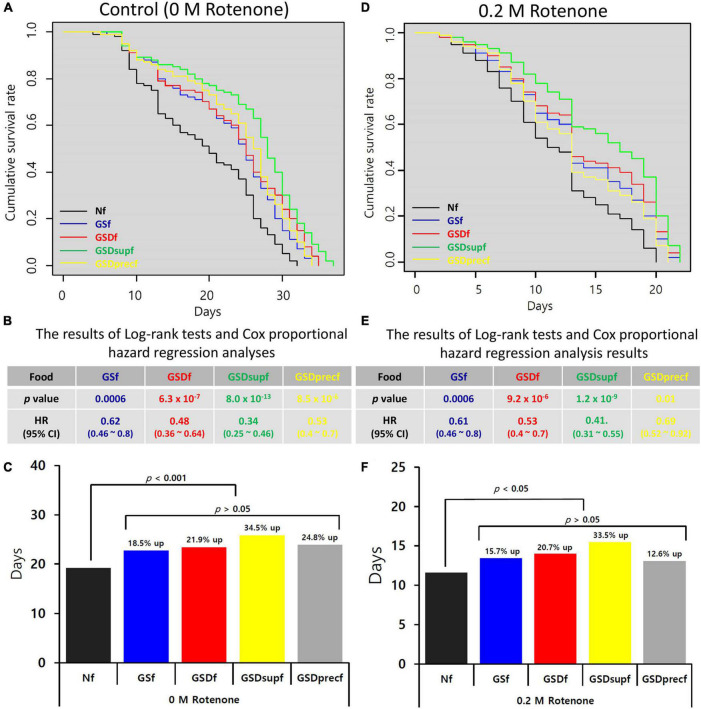
Prevention of the onset and progression of rotenone-induced loss of motor control by GS, GSD, GSDsup, or GSDprec. **(A)** The lifespans of GSf, GSDf, GSDsupf, or GSDprecf-reared *Drosophila* were extended compared with that of Nf-reared *Drosophila.* **(B)** In comparison with Nf-reared *Drosophila*, the HRs of *Drosophila* reared with GSf, GSDf, GSDsupf, or GSDprecf were significantly reduced, when they were not treated with rotenone. **(C)** The average lifespans of GSf-, GSDf-, GSDsupf-, or GSDprecf-reared *Drosophila* were more promoted than that of Nf-reared *Drosophila*. **(D)** When *Drosophila* was exposed to 0.2 M rotenone, the lifespans of GSf, GSDf, GSDsupf, or GSDprecf-reared *Drosophila* were extended compared with that of Nf-reared *Drosophila.* **(E)** The HRs of GSf-, GSDf-, GSDsupf-, and GSDprecf-reared *Drosophila* were significantly reduced. **(F)** The average lifespans of GSf-, GSDf-, GSDsupf-, and GSDprecf-reared *Drosophila* were more promoted than that of Nf-reared *Drosophila*.

Rotenone is a widely used natural plant protection agent that is extracted from the roots of *Derris* spp., *Lonchocarpus* spp., *Tephrosia* spp., and *Mundulea* spp. Although rotenone is widely used in eco-friendly or organic farming, an important side effect observed in humans and animals exposed long-term to rotenone is loss of motor control because this compound is a MitoCom I inhibitor (39). In previous studies, we showed that GS could prevent the onset and progression of rotenone-induced loss of motor control in *Drosophila* (13, 14). Thus, we tested whether GSD, GSDsup, or GSDprec could prevent the onset and progression of loss of motor control in *Drosophila* (Figure 3). In addition to GSf-reared *Drosophila* (HR = 0.61), the HRs of GSDf- (0.48), GSDsupf- (0.34), and GSDprecf-reared *Drosophila* (0.53) were significantly reduced (*p* < 0.001, Figures 3A,B). Compared with that of Nf-reared *Drosophila* (19.15 days), the average lifespans of GSf-, GSDf-, GSDsupf-, and GSDprecf-reared *Drosophila* were 22.69 days (18.5% up), 23.34 days (21.9% up), 25.75 days (34.5% up), and 23.89 days (24.8% up), respectively (Figure 3C). When *Drosophila* was exposed to 0.2 M rotenone, compared with Nf-reared *Drosophila*, the HRs of GSf- (0.61), GSDf- (0.53), GSDsupf- (0.41), and GSDprecf-reared *Drosophila* (0.69) were significantly reduced (*p* < 0.001, Figures 3D,E). Compared with that of Nf-reared *Drosophila* exposed to 0.2 M rotenone (11.58 days), the average lifespans of GSf-, GSDf-, GSDsupf-, or GSDprecf-reared *Drosophila* exposed to 0.2 M rotenone were 13.4 days (15.7% up), 13.98 days (20.7% up), 15.46 days (33.5% up), and 13.04 days (12.6% up), respectively (Figure 3F). This result suggested that GSDsupf-reared *Drosophila* exhibited the superior prevention effect of rotenone-induced loss of motor control.

### More Total Phenolic Compounds and Antioxidant Activities in GSD and GSDsup

**FIGURE 4:**
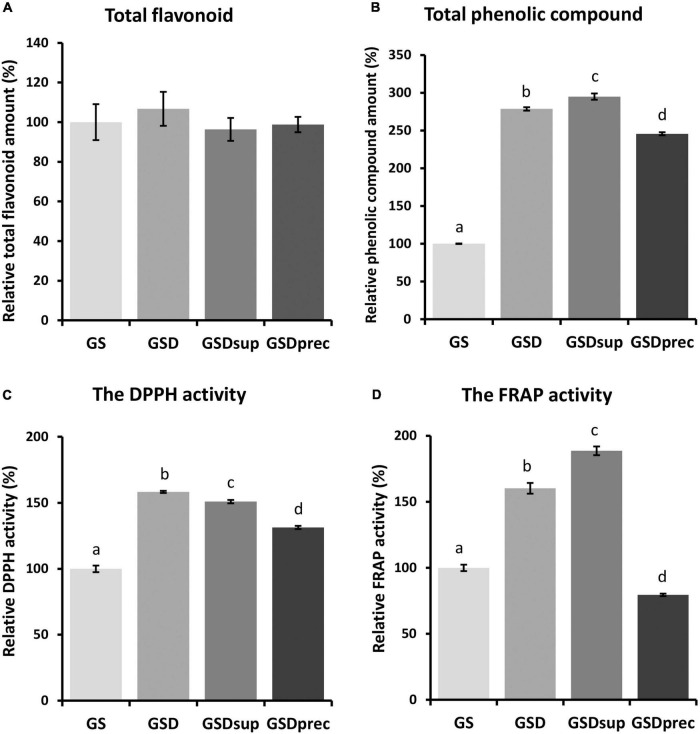
The amounts of phytochemicals and antioxidant activities of 80% methyl alcohol (MeOH) extracts of GS, GSD, GSDsup, and GSDprec. **(A)** There were non-significant differences in the amounts of total flavonoids among the 80% MeOH extracts of GS, GSD, GSDsup, and GSDprec [*F*_(3,19)_ = 0.38698, *p* = 0.76]. **(B)** There were significant differences in the amounts of total phenolic compounds among the 80% MeOH extracts of GS, GSD, GSDsup, and GSDprec [*F*_(3,19)_ = 1229.14, *p* = 4.0 × 10^– 19^]. **(C)** There were significant differences in the 1,1-di-phenyl-2-picryl-hydrazyl (DPPH) radical scavenger activities among the 80% MeOH extracts of GS, GSD, GSDsup, and GSDprec [*F*_(3,19)_ = 261.98, *p* = 8.3 × 10^– 14^]. **(D)** There were significant differences in the ferric-reducing ability of plasma (FRAP) activities among the 80% MeOH extracts of GS, GSD, GSDsup, and GSDprec [*F*_(3,19)_ = 304.9, *p* = 2.5 × 10^– 14^]. The letters above the error bars represent significant differences as determined by one-way ANOVA and Tukey’s HSD *post-hoc* test.

To investigate the effect of FP^®^ AP treatment on the quantity and antioxidant activity of phytochemicals and small molecules present in GS, 80% MeOH extracts of GS, GSD, GSDsup, and GSDprec were used. There were non-significant differences in the total flavonoid amounts in the 80% MeOH extracts (GS = 100.0 ± 9.07%, GSD = 106.7 ± 8.57%, GSDsup = 96.3 ± 5.79, GSDprec = 98.8 ± 3.90, *p* > 0.05, Figure 4A). By contrast, the relative total phenolic compounds of GSD (278.7 ± 2.25%), GSDsup (295.0 ± 4.06%), and GSDprec (245.7 ± 2.01%) were significantly increased compared to those of GS (100.0 ± 0.43%) (*p* < 0.005, Figure 4B).

The antioxidant effects of the GS, GSD, GSDsup, and GSDprec extracts were examined by using DPPH and FRAP assays. The DPPH radical scavenging activity was significantly increased with GSD extract (158.3 ± 0.82%), GSDsup extract (150.9 ± 1.30%), and GSDprec extract (131.3 ± 1.23%) compared with GS extract (100 ± 2.54%) (*p* < 0.005, Figure 4C). By contrast, the FRAP activities were significantly increased in the GSD extract (160.2 ± 4.05%) and GSDsup extract (188.6 ± 3.27%) but decreased significantly with the GSDprec extract (79.4 ± 0.91%) compared to the GS extract (100.0 ± 2.49%) (*p* < 0.005, Figure 4D). These results suggest that the increased antioxidant effect observed in the GSD extract is probably due to the increased phenolic compounds in the GSDsup extract. In addition, the increased amount of total phenolic compounds and antioxidant effect in GSD, GSDsup, and GSDprec compared to GS (Figure 4) might be used together with FPLC chromatograms (Supplementary Figure 1) to assure the quality of the samples.

## Discussion

More than 1,900 species of edible insects worldwide are consumed as food and feed that supplies protein and lipids (40–42). Compared to traditional livestock, edible insects called mini-livestock have epidemiological advantages for use as foods and feeds, because there are no common infectious disease pathogens between insects and humans or vertebrates. By contrast, feeding livestock with the same or other species byproducts is known to cause severe diseases that can be transmitted to humans. More than 60% of human diseases originate from vertebrate animals, including domestic livestock, pets, and wild animals (43). Therefore, edible insects can be used as a portion of major food and feed sources to cope with infectious diseases that threaten humanity now and in the future. Furthermore, in addition to being used as food or feed, certain edible insects may have special health-promoting effects. One of the most important examples is the mulberry silkworm, the longest-reared mini-livestock by humans (1, 2). Prior to the 1990s, silkworm pupae, a byproduct of the process of producing silk fibers from cocoons, were primarily used as protein and fat sources. By scientifically investigating the health-promoting effects of various sericulture products mentioned in ancient Oriental medical documents since the 1990s, various health enhancement effects of silkworm products have been revealed (1, 2, 42). People can eat meats, poultry, fish, eggs, etc., which are products of traditional livestock without any aversion. However, there are great preference differences among people for edible insects. Thus, research has been conducted toward producing edible insects as general food, health functional food, or food for special medical purposes after reducing aversion due to shape or smell through various treatment processes rather than the raw materials (2).

The advantage of silkworms compared to other edible insects is that their body wall is very thin and soft, so they can be consumed whole by steaming and freeze-drying (9). In previous studies, we have shown that the size of the final powder is related to the health-promotion effects of HongJam. When a hammer mill, a natural stone roller mill, or an air-jet mill were used, the average size of the final powders that could be obtained was approximately 120 μm, 10 μm, or 1 μm, respectively (9, 30, 31). Although there was a significant difference in the health-promotion effects and contents of nutrients between HongJam powder sizes of 120 μm and 10 μm (30), there was no significant difference in the contents of nutrients between HongJam powder sizes of 10 μm and 1 μm (19). The reason that there were no differences in the health-promotion effects and the nutritional compositions between HongJam powder sizes of 10 μm and 1 μm is probably that the powders containing fatty acids are lost because of the mechanical characteristics of an air jet mill used for pulverizing. These results suggested it is necessary to develop a method of making the size of HongJam powders smaller without the loss of certain nutrients or transforming them into a form that might be easily absorbed by humans or animals. Therefore, the most important contribution from this study was providing the molecular and biochemical evidence of how the treatment of food-grade protease enhances the health-promotion effects of HongJam powder with a size of 10 μm.

Interestingly, we found that the effect of food-grade protease treatment on HongJam was different depending on the silkworm variety when comparing the healthspan-promoting effect. There was no difference in the healthspan-promoting effect between WJ and WJD made with the white-colored cocoon silkworm variety, while a significant healthspan-promoting effect was present in GSD compared to GS made with the yellow-colored cocoon silkworm variety (Figure 1). In a previous study, we reported that GS had a superior memory improvement effect in mild cognitive impairment rodent models compared to WJ (11). Since we reported that the memory improvement effect in the mild cognitive impairment model by GS was due to the enhancement of mitochondrial functions, the enhancement of mitochondrial functions of GS- and GSD-reared *Drosophila* was compared in this study (Figure 2). However, unexpectedly, there was no significant difference in the mitochondrial function-enhancing effect between GS and GSD. These results suggested the possibility that the healthspan-promoting effect of GSD can be achieved by activating certain signal transduction pathways in addition to mitochondrial function enhancement. To support this hypothesis, we conducted a study on the signal transduction mechanisms related to healthspan promotion or disease inhibition, such as Tor, autophagy, and UPR signaling (44). As expected, we confirmed that the expression of autophagy- and UPR-associated genes increased more in GSDf-reared *Drosophila* (Figures 3, 4). This result was further reinforced by the increased resistance of GSDf-, GSDsupf-, and GSDprecf-reared *Drosophila* to the autophagy inhibitor 3-MA (Table 1).

Another important finding from this study was that GSDsup had an excellent inhibitory effect on the onset of rotenone-induced loss of motor control compared to GS, GSD, or GSDprec (Figure 3). The reason this result is important is that, for proteases, large molecular weight–proteins that make up silk fibers, such as Fibroin, may be degraded into small molecular weight peptides, and phytochemicals that are strongly bound to the silk fibers may be dissociated and then rapidly absorbed into the body, resulting in excellent healthspan promotion and inhibition of the onset of rotenone-induced loss of motor control. Consistent with our hypothesis, we confirmed in this study that GSDsup had the highest total polyphenol content and antioxidant activity (Figure 4) and more small molecular weight peptides than GS, GSD, or GSDprec (Supplementary Figure 1). In addition, we have shown that digestion of freeze-dried 5th instar 3rd-day larval powders with food-grade proteases increased small molecular weight peptides in the previous study (33), supporting our results.

Interestingly, recent studies have reported that phytochemicals in food inhibit the onset of PD by activating autophagy signaling (29, 45–47). In a previous study, we reported HongJam contains significant amounts of phytochemicals such as rutin, quercetin, isoquercetin, kaempferol, and astragalin (14, 36). Since quercetin glycosides (47), kaempferol (45, 48), or astragalin (49) are reported to have inhibitory effects on the onset of PD in rodent models and/or cellular models, it is speculated that various types of functional nutrients with small molecular weights present in GSDsup are more rapidly absorbed into the body and delivered to the brain, thereby exhibiting excellent inhibitory effects during the onset of rotenone-induced loss of motor control.

In summary, the reason GSDsup was more effective in healthspan promotion and prevention of the onset of rotenone-induced loss of motor control compared to other samples is that GSDsup may contain more free phytochemicals and small molecular weight peptides that enhance autophagy signaling and mitochondrial function of *Drosophila* than other samples. Nevertheless, further clinical and preclinical researches are needed to confirm the prevention or treatment of PD through activation of autophagy signaling by phytochemicals or other small molecular weight molecules in GSDsup.

## Data Availability Statement

The original contributions presented in the study are included in the article/Supplementary Material, further inquiries can be directed to the corresponding author/s.

## Author Contributions

LXM, S-KK, Y-YJ, and PN performed experiments, analyzed data, and made Figures 1–4, Table 1, and Supplementary Data. A-YK, K-YK, and N-SK provided HongJam, performed experiments, and wrote manuscript. YHK designed and conceived experiments, got funded, analyzed data, and wrote manuscript. All authors reviewed the manuscript and approved the submitted version.

## Conflict of Interest

The authors declare that the research was conducted in the absence of any commercial or financial relationships that could be construed as a potential conflict of interest.

## Publisher’s Note

All claims expressed in this article are solely those of the authors and do not necessarily represent those of their affiliated organizations, or those of the publisher, the editors and the reviewers. Any product that may be evaluated in this article, or claim that may be made by its manufacturer, is not guaranteed or endorsed by the publisher.

## Footnotes

1
https://string-db.org/

## References

[1] Koh YH. The memory enhancement and healthspan extension effects of HongJam. *Policy Rep Natl Inst Korean Med Dev.* (2020) 5:22–33. 10.1016/j.jep.2021.114520 34391862

[2] Kim KY Osabutey AF Nguyen P Kim SB Jo YY Kweon HY The experimental evidences of steamed and freeze-dried mature silkworm powder as the calorie restriction mimetics. *Int J Indust Entomol.* (2019) 39:1–8. 10.7852/ijie.2019.39.1.1

[3] Ryu KS Lee HS Chung SH Kang PD. An activity of lowering blood-glucose levels accoring to preparative conditions of silkworm powder. *J Seric Entomol Sci.* (1997) 39:79–85.

[4] Ryu KS Lee HS Kim KY Kim MJ Kang PD Chun SN Anti-diabetic effects of the silkworm (*Bombyx mori*.) extracts in the db/db mice. *Planta Med.* (2012) 78:I458. 10.1055/s-0032-1321145

[5] Ryu KS Lee HS Kim KY Kim MJ Sung GB Ji SD 1-deoxynojirimycin content and blood glucose-lowering effect of silkworm (*Bombyx mori*) extract powder. *Int J Indust Entomol.* (2013) 27:237–42. 10.7852/ijie.2013.27.2.237

[6] Oh HG Lee HY Kim JH Kang YR Moon DI Seo MY Effects of male silkworm pupa powder on the erectile dysfunction by chronic ethanol consumption in rats. *Lab Anim Res.* (2012) 28:83–90. 10.5625/lar.2012.28.2.83 22787481 PMC3389843

[7] Kang YK Lee BY Bucci LR Stohs SJ. Effect of a Fibroin enzymatic hydrolysate on memory improvement: a placebo-controlled, double-blind study. *Nutrients.* (2018) 10:233. 10.3390/nu10020233 29462997 PMC5852809

[8] Kang YK Lee WJ Kang BH Kang HN. Memory-enhancing fffects of silk fibroin-derived peptides in scopolamine-treated mice. *J Microbiol Biotechn.* (2013) 23:1779–84. 10.4014/jmb.1308.08059 24043122

[9] Ji SD Kim NS Lee JY Kim MJ Kweon H Sung G Development of processing technology for edible mature silkworm. *J Seric Entomol Sci.* (2015) 53:38–43. 10.7852/jses.2015.53.1.38

[10] Ji SD Nguyen P Yoon SM Kim KY Son JG Kweon HY Comparison of nutrient compositions and pharmacological effects of steamed and freeze-dried mature silkworm powders generated by four silkworm varieties. *J Asia Pacific Entomol.* (2017) 20:1410–8. 10.1016/j.aspen.2017.10.010

[11] Nguyen P Kim KY Kim AY Choi BH Osabutey AF Park YH Mature silkworm powders ameliorated scopolamine-induced amnesia by enhancing mitochondrial functions in the brains of mice. *J Funct Foods.* (2020) 67:103886. 10.1016/j.jff.2020.103886

[12] Nguyen P Kim KY Kim AY Kang S Osabutey AF Jin H The additive memory and healthspan enhancement effects by the combined treatment of mature silkworm powders and Korean angelica extracts. *J Ethnopharmacol.* (2021) 281:114520. 34391862 10.1016/j.jep.2021.114520

[13] Nguyen P Kim KY Kim AY Kim NS Kweon H Ji SD Increased healthspan and resistance to Parkinson’s disease in *Drosophila* by boiled and freeze-dried mature silk worm larval powder. *J Asia Pacific Entomol.* (2016) 19:551–61. 10.1016/j.aspen.2016.05.003

[14] Choi BH Ji SD Son JG Nguyen P Kim KY Park YH Phytochemicals and silk proteins in mature silkworm powders responsible for extended life expectancy and enhanced resistances to Parkinson’s disease. *J Asia Pacific Entomol.* (2017) 20:1425–33. 10.1016/j.aspen.2017.10.011

[15] Ji SD Son JG Koh YH. Some skeletons in the mature silkworm larvae: not only spinning silk threads but also preventing Parkinson’s disease. *J Alzheimers Dis Parkisonism.* (2016) 6:7. 10.4172/2161-0460.1000289

[16] Lee DY Cho JM Yun SM Hong KS Ji SD Son JG Comparative effect of silkworm powder from 3 *Bombyx mori* varieties on ethanol-induced gastric injury in rat model. *Int J Indust Entomol.* (2017) 35:14–21. 10.7852/ijie.2017.35.1.14

[17] Yun SM Cho JM Hong KS Lee DY Ji SD Son JG Gastroprotective effect of mature silkworm, *Bombyx mori* against ethanol-induced gastric mucosal injuries in rats. *J Funct Foods.* (2017) 39:279–86. 10.1016/j.jff.2017.10.036

[18] Hong KS Yun SM Cho JM Lee DY Ji SD Son JG Silkworm (*Bombyx mori*) powder supplementation alleviates alcoholic fatty liver disease in rats. *J Funct Foods.* (2018) 43:29–36. 10.1016/j.jff.2018.01.018

[19] Ji SD Kim SB Kim KY Kim NS Kim SW Jo YY Contents of nutrients in ultra-fine powders of steamed and lyophilized mature silkworms generated by four silkworm varieties. *J Asia Pacific Entomol.* (2019) 22:969–74. 10.1016/j.aspen.2019.07.009

[20] Proshkina EN Shaposhnikov MV Sadritdinova AF Kudryavtseva AV Moskalev AA. Basic mechanisms of longevity: a case study of *Drosophila* pro-longevity genes. *Ageing Res Rev.* (2015) 24:218–31. 10.1016/j.arr.2015.08.005 26318059

[21] Piper MDW Partridge L. *Drosophila* as a model for ageing. *Biochim Biophys Acta Mol Basis Dis.* (2018) 1864:2707–17. 10.1016/j.bbadis.2017.09.016 28964875

[22] Bansal A Zhu LJ Yen K Tissenbaum HA. Uncoupling lifespan and healthspan in *Caenorhabditis elegans* longevity mutants. *Proc Nat Acad Sci USA.* (2015) 112:E277–86. 10.1073/pnas.1412192112 25561524 PMC4311797

[23] Koh YH. *Drosophila* as a model organism for investigating molecular and cellular etiologies underlying complex neurological disorders in humans. *J Asia Pacific Entomol.* (2006) 9:75–84.

[24] Gaitanidis A Dimitriadou A Dowse H Sanyal S Duch C Consoulas C. Longitudinal assessment of health-span and pre-death morbidity in wild type *Drosophila*. *Aging.* (2019) 11:1850–73. 10.18632/aging.101880 30923256 PMC6461171

[25] Blesa J Przedborski S. Parkinson’s disease: animal models and dopaminergic cell vulnerability. *Front Neuroanat.* (2014) 8:155. 10.3389/fnana.2014.00155 25565980 PMC4266040

[26] Whitworth AJ. *Drosophila* models of Parkinson’s disease. *Adv Genet.* (2011) 73:1–50. 10.1016/B978-0-12-380860-8.00001-X 21310293

[27] Duty S Jenner P. Animal models of Parkinson’s disease: a source of novel treatments and clues to the cause of the disease. *Br J Pharmacol.* (2011) 164:1357–91. 10.1111/j.1476-5381.2011.01426.x 21486284 PMC3229766

[28] Hou X Watzlawik JO Fiesel FC Springer W. Autophagy in Parkinson’s disease. *J Mol Biol.* (2020) 432:2651–72. 10.1016/j.jmb.2020.01.037 32061929 PMC7211126

[29] Balakrishnan R Azam S Cho DY Kim IS Choi DK. Natural phytochemicals as novel therapeutic strategies to prevent and treat Parkinson’s disease: current knowledge and future perspectives. *Oxid Med Cell Longev.* (2021) 2021:6680935. 10.1155/2021/6680935 34122727 PMC8169248

[30] Ji SD Son JG Kim S Kim NS Kim KY Kweon H Production techniques to improve the quality of steamed and freeze-dried mature silkworm larval powder. *Int J Indust Entomol.* (2017) 34:1–11. 10.7852/ijie.2017.34.2.17

[31] Kim SB Kim KY Ji SD Kim SW Kim NS Jo YY Effect of pulverizing method on the particle size of matured silkworm powder. *Int J Indust Entomol.* (2018) 37:105–8. 10.7852/ijie.2018.37.2.105

[32] Ji SD Kim NS Kweon H Choi BH Yoon SM Kim KY Nutrient compositions of *Bombyx mori* mature silkworm larval powders suggest their possible health improvement effects in humans. *J Asia Pacific Entomol.* (2016) 19:1027–33. 10.1016/j.aspen.2016.08.004

[33] Joung JA Park MN You JY Song BJ Choi JH. Application of food-grade proteolytic enzyme for the hydrolysis of regenerated silk fibroin from *Bombyx mori*. *J Chem.* (2018) 2018:1285823. 10.1155/2018/1285823

[34] Bae SM Jo YY Lee KG Kim HB Kweon H. Antioxidant activity of silkworm powder treated with protease. *Int J Indust Entomol.* (2016) 33:78–84. 10.7852/ijie.2016.33.2.78

[35] Szklarczyk D Gable AL Nastou KC Lyon D Kirsch R Pyysalo S The STRING database in 2021: customizable protein-protein networks, and functional characterization of user-uploaded gene/measurement sets. *Nucleic Acids Res.* (2021) 49:D605–12. 10.1093/nar/gkaa1074 33237311 PMC7779004

[36] Choi BH Ji SD Jeong JH Kim KY Koh YH. Quantification and comparison of functional phytochemicals in steamed and freeze-dried mature silkworm powders and freeze-dried mulberry leaves. *Int J Indust Entomol.* (2017) 35:89–96. 10.7852/ijie.2017.35.2.89

[37] Kim AY Seo JB Kim WT Choi HJ Kim SY Morrow G The pathogenic human torsin A in *Drosophila* activates the unfolded protein response and increases susceptibility to oxidative stress. *BMC Genomics.* (2015) 16:338. 10.1186/s12864-015-1518-0 25903460 PMC4415242

[38] Hansen M Kennedy BK. Does longer lifespan mean longer healthspan? *Trends Cell Biol.* (2016) 26:565–8. 10.1016/j.tcb.2016.05.002 27238421 PMC4969078

[39] Gupta RC. Rotenone. 3rd ed. In: Wexler P editor. *Encyclopedia of Toxicology.* (Oxford: Academic Press) (2014). 10.1016/B978-0-12-386454-3.00194-9

[40] van Huis A Itterbeeck JV Klunder H Mertens E Halloran A Muir G *Edible Insects Future Prospects for Food and Feed Security.* Rome: Food and Agriculture Organization of the United Nations (2013).

[41] van Huis A. Potential of insects as food and feed in assuring food security. *Ann Rev Entomol.* (2013) 58:563–83. 10.1146/annurev-ento-120811-153704 23020616

[42] Park SJ Kim KY Baik MY Koh YH. Sericulture and the edible-insect industry can help humanity survive: insects are more than just bugs, food, or feed. *Food Sci Biotechnol.* (2022) 31:657–668. 10.1007/s10068-022-01090-3 35646418 PMC9133288

[43] Jones KE Patel NG Levy MA Storeygard A Balk D Gittleman JL Global trends in emerging infectious diseases. *Nature.* (2008) 451:990–3. 10.1038/nature06536 18288193 PMC5960580

[44] Aman Y Schmauck-Medina T Hansen M Morimoto RI Simon AK Bjedov I Autophagy in healthy aging and disease. *Nat Aging.* (2021) 1:634–50. 10.1038/s43587-021-00098-4 34901876 PMC8659158

[45] Pan X Liu X Zhao H Wu B Liu G. Antioxidant, anti-inflammatory and neuroprotective effect of kaempferol on rotenone-induced Parkinson’s disease model of rats and SH-S5Y5 cells by preventing loss of tyrosine hydroxylase. *J Funct Foods.* (2020) 74:104140. 10.1016/j.jff.2020.104140

[46] Tamtaji OR Hadinezhad T Fallah M Shahmirzadi AR Taghizadeh M Behnam M The therapeutic potential of quercetin in Parkinson’s disease: insights into its molecular and cellular regulation. *Curr Drug Targets.* (2020) 21:509–18. 10.2174/1389450120666191112155654 31721700

[47] Magalingam KB Radhakrishnan A Ramdas P Haleagrahara N. Quercetin glycosides induced neuroprotection by changes in the gene expression in a cellular model of Parkinson’s disease. *J Mol Neurosci.* (2015) 55:609–17. 10.1007/s12031-014-0400-x 25129099

[48] Calderon-Montaño JM Burgos-Moron E Perez-Guerrero C Lopez-Lazaro M. A review on the dietary flavonoid kaempferol. *Mini Rev Med Chem.* (2011) 11:298–344. 10.2174/138955711795305335 21428901

[49] Riaz A Rasul A Hussain G Zahoor MK Jabeen F Subhani Z Astragalin: a bioactive phytochemical with potential therapeutic activities. *Adv Pharmacol Sci.* (2018) 2018:9794625. 10.1155/2018/9794625 29853868 PMC5954929

