# Supplementary material for: An Alkaline Protease-Digestion of Silkworm Powder Enhances Its Effects Over Healthspan, Autophagy, and Mitochondria Function in a Rotenone-Induced Drosophila Model
Source: Front Nutr. 2022 Jun 16;9:808295. doi: 10.3389/fnut.2022.808295 (PMC9244169; doi:10.3389/fnut.2022.808295)

Supplementary Material

# Supplementary Tables

Supplementary Table 1. DNA sequences of oligomers and running conditions of RT-qPCRs used in this study

| Signaling | Gene | Oligomer sequences (F: forward/R: reverse) | | PCR amplification protocol |
| --- | --- | --- | --- | --- |
| Loading  control | RP49 | F | CAGTCGGATCGATATGCTAAGCTGT | 95^o^C-3 min, 1 cycle; 95^o^C-5 sec, 62 ^o^C-10 sec, 72 ^o^C-10 sec, 40 cycles |
|  |  | R | TAACCGATGTTGGGCATCAGATACT |  |
| UPR  (Kim et al, 2015) | Hsc70-3 | F | GCTCAACCTGGATCTATTCC |  |
|  |  | R | TGGATGGTGACGGTGTGCTGGTTAT |  |
|  | Hsc70-4 | F | AGGTGTACGAGGGAGAGCGT |  |
|  |  | R | CCACTTGATGGTCTCGTTGC |  |
|  | Hsc70-5 | F | GAGCGTGAGATGGCTAACGA |  |
|  |  | R | AGGTCGGCAAGTCTCCTTGTT |  |
|  | Ire1 | F | GATCCAATGGAAGCACTGGCAGCAA |  |
|  |  | R | TTGTGCTGAAGCTGATCTTGCCCAC |  |
|  | PEK | F | TCTGGTCATTGAACGTCATGTGCCTG |  |
|  |  | R | TGATTTGCTTGTCCAGGTGGGAAGC |  |
|  | Atf6 | F | AACGTAATTCCACGGAAGCCCAACA |  |
|  |  | R | GCGACGGTAGCTTGATTTCTAGAGCC |  |
|  | Xbp1^+23bp^ | F | GCACAACACCAGATGCATCAGCCAA |  |
|  |  | R | AGAGGGTCAGCTTTGGATGCTGCAGA |  |
|  | Xbp1^-23bp^ | F | GCACAACACCAGATGCATCAGCCAA |  |
|  |  | R | TGTTGTATACCCTGCGGCAGATCCAA |  |
|  | crc | F | TGCGAGTCTCAGGCGTCTTCATCTT |  |
|  |  | R | CTGCTCGATGGTTGTAGGAGCTGG |  |
|  | GP93 | F | TACCTGAGCTTCATTCGTGGCGTCG |  |
|  |  | R | GCGGACCAGCTTCTTCTTGATCACC |  |
|  | GRP170 | F | CAGTCTGGACGTGATCAACCAGGTG |  |
|  |  | R | ACTGCACCCATAGTGGCGGATTCAT |  |
| Autophagy  Signaling  (Kim et al, 2015) | Atg1 | F | GGATCATTGGGCTCGATTGGTTCGG |  |
|  |  | R | GGCTCCTGTGTCCAGCAGACTATG |  |
|  | Atg2 | F | TTCACGCATGGACAAGTTCGGTGTC |  |
|  |  | R | GATGGAGGCCTAAGCAGGTCCACTT |  |
|  | Atg3 | F | CAAGCCACTGACCGTGGAACAGATG |  |
|  |  | R | CACAGAGGCCATATTGGGACCTGGA |  |
|  | Atg4a | F | CGACTTACATTTGGGACGCGACTGG |  |
|  |  | R | ACTCTCCCATCTGGGCAATCTGGTG |  |
|  | Atg5 | F | TGGTGCCTCACCATTCACTTCTCCA |  |
|  |  | R | TGCCTCCTTCAGGCAGGACATGTAG |  |
|  | Atg6 | F | CAGATCGCCTACTCGAAACAGCAGC |  |
|  |  | R | AGTCCACGGATACAGAGGGCAATCG |  |
|  | Atg7 | F | CGACATGGCACTACGCGTAAGGAAG |  |
|  |  | R | CGGTCTTTGAGCGAATTTCCAGGGG |  |
|  | Atg8a | F | CTCAGCAACGCATCGATATTCGGCA |  |
|  |  | R | CGAAGGCGTGCTCCTCCTTGTATTG |  |
|  | Atg8b | F | CCGATGAGAACGTCTATGGACGGCA |  |
|  |  | R | CTGGTTAGCTGGCTAGGAGCAGTCA |  |
|  | Atg9 | F | ACTGCTCTACGGACTCTGTGGTGTG |  |
|  |  | R | GTGCAAATAGAGGGCACTGAGGCAC |  |
|  | Atg12 | F | ACAGTCGGCTGGATACAGACGTTCA |  |
|  |  | R | TCCCATTGGTTCCATGGCACTCGTA |  |
|  | Atg13 | F | GTGGTGGGCTCCAAATGAGCAACTG |  |
|  |  | R | GCCTCTCTCCATATTGACCGCCCTT |  |
|  | Atg18a | F | TTGATTGCCTCGCAGGATGGGTACT |  |
|  |  | R | GCCGCAGGTCGTGCCTCTTTATTAG |  |
| Tor signaling | AKT1 | F | CCGCAGTGCAGTAGGGAAAGCAAA |  |
|  |  | R | GCGACCTAGCAGCGCAACATGT |  |
|  | S6K | F | ACTGGGCGCTCTCATGTTTG |  |
|  |  | R | TTGGCTTTCAGAATGGTC |  |
|  | Tor | F | GAGTGAGTTCCTGGCTCCCCGG |  |
|  |  | R | GGGCGAAATGCCCTTCCTGGC |  |
|  | PI3K | F | GCTATAAAGGCGAGGGCTGGCC |  |
|  |  | R | ACGCGGCACCGATGTCTCATC |  |
|  | foxo | F | AGGCGCAGCCGATAGACGAATTTA |  |
|  |  | R | TGCTGTTGACCAGGTTCGTGTTGA |  |
| Gustatory receptors | Gr64a | F | GTGTGCTACCAACTGCTAAATGTC |  |
|  |  | R | ACCTCGTTTGGACTCCTCA |  |
|  | Gr64b | F | CTATCGGTTCTACGGCGAGTAC |  |
|  |  | R | ACTGGGTGCGCTCCATATTG |  |
|  | Gr64c | F | CTCAGTGATCTTCTGGGTATCTACG |  |
|  |  | R | ATCCGCATAGTTGCCCTTGG |  |
|  | Gr64d | F | TGCTTCGCAATGAAACCTTTGC |  |
|  |  | R | CTTGCATTTGCCGGAACAGC |  |
|  | Gr64e | F | ACCTTCGCCTGGAACTTTAACG |  |
|  |  | R | CCTGCACTGCATCCAATAGTCC |  |
|  | Gr64f | F | CCGCAGTACAAGACACAGTTGAC |  |
|  |  | R | TCCGCAAAGGACAGCATCATTC |  |

Supplementary Table 2. Differentially expressed genes in AKT/PI3K/Tor-, UPR-, and autophagy-signaling and 6 Grs in the heads and bodies of 7-day-old Nf-, GSf-, or GSDf-reared *Drosophila*

| **Genes** | | **Head** | | | **Body** | | |
| --- | --- | --- | --- | --- | --- | --- | --- |
|  |  | **Nf** | **GSf** | **GSDf** | **Nf** | **GSf** | **GSDf** |
| **UPR** | **Hsc70-3** | 1.0 ± 0.069^a^ | 0.59 ± 0.029^b^ ↓ | 0.35 ± 0.043^c^ ↓ | 1.0 ± 0.077^a^ | 0.58 ± 0.072^b^ ↓ | 0.93 ± 0.052^a^ |
|  | **Ire1** | 1.0 ± 0.093^a^ | 1.21 ± 0.137^a^ | 0.94 ± 0.063^a^ | 1.0 ± 0.082^a^ | 0.64 ± 0.132^b^ ↓ | 1.48 ± 0.095^c^ ↑ |
|  | **PEK** | 1.0 ± 0.084^a^ | 1.24 ± 0.207^a^ | 0.35 ± 0.011^b^ ↓ | 1.0 ± 0.068^a^ | 0.30 ± 0.133^b^ ↓ | 1.26 ± 0.038^a^ |
|  | **ATF6** | 1.0 ± 0.078^a^ | 0.68 ± 0.043^b^ ↓ | 0.55 ± 0.080^a^ ↓ | 1.0 ± 0.071^a^ | 1.03 ± 0.079^a^ | 1.67 ± 0.120^b^ ↑ |
|  | **Xbp1^+23bp^** | 1.0 ± 0.090^a^ | 0.59 ± 0.038^b^ ↓ | 0.57 ± 0.027^b^ ↓ | 1.0 ± 0.059^a^ | 0.41 ± 0.079^b^ ↓ | 0.55 ± 0.024^b^ ↓ |
|  | **Xbp1^-23bp^** | 1.0 ± 0.060^a^ | 0.94 ± 0.049^a^ | 0.60 ± 0.025^b^ ↓ | 1.0 ± 0.152^a^ | 0.97 ± 0.054^a^ | 0.79 ± 0.059^a^ |
|  | **crc** | 1.0 ± 0.012^a^ | 0.79 ± 0.097^b^ ↓ | 0.66 ± 0.036^b^ ↓ | 1.0 ± 0.067^a^ | 0.58 ± 0.067^a^ ↓ | 0.94 ± 0.0.64^a^ |
|  | **Grp-170** | 1.0 ± 0.011^a^ | 0.92 ± 0.166^a^ | 0.75 ± 0.038^a^ | 1.0 ± 0.119^a^ | 0.66 ± 0.275^a^ | 1.46 ± 0.136^a^ |
|  | **Gp93** | 1.0 ± 0.038^a^ | 0.42 ± 0.064^b^ ↓ | 0.59 ± 0.041^a^ ↓ | 1.0 ± 0.077^a^ | 0.58 ± 0.072^b^ ↓ | 0.93 ± 0.052^a^ |
| **Autophagy** | **Hsc70-4** | 1.0 ± 0.086^a^ | 0.53 ± 0.09^b^ ↓ | 0.50 ± 0.030^b^ ↓ | 1.0 ± 0.094^a^ | 0.22 ± 0.056^b^ ↓ | 0.50 ± 0.030^c^ ↓ |
|  | **Hsc70-5** | 1.0 ± 0.071^a^ | 0.8 ± 0.099^a^ | 0.81 ± 0.146^a^ | 1.0 ± 0.086^a^ | 0.57 ± 0.166^b^ ↓ | 0.91 ± 0.098^a^ |
|  | **Atg1** | 1.0 ± 0.129^a^ | 0.62 ± 0.068^b^ ↓ | 0.62 ± 0.062^b^ ↓ | 1.0 ± 0.092^a^ | 0.57 ± 0.105^b^ ↓ | 0.56 ± 0.051^b^ ↓ |
|  | **Atg2** | 1.0 ± 0.050^a^ | 0.45 ± 0.088^b^ ↓ | 0.96 ± 0.149^a^ | 1.0 ± 0.123^a^ | 0.32 ± 0.141^b^ ↓ | 1.04 ± 0.047^a^ |
|  | **Atg3** | 1.0 ± 0.038^a^ | 0.96 ± 0.021^a^ | 0.80 ± 0.124^a^ | 1.0 ± 0.034^a^ | 1.48 ± 0.036^b^ ↑ | 1.30 ± 0.167^ab^ |
|  | **Atg4a** | 1.0 ± 0.091^a^ | 0.56 ± 0.052^b^ ↓ | 0.71 ± 0.066^b^ ↓ | 1.0 ± 0.061^a^ | 0.77 ± 0.068^b^ ↓ | 1.01 ± 0.042^a^ |
|  | **Atg5** | 1.0 ± 0.074^a^ | 0.74 ± 0.022^b^ ↓ | 0.70 ± 0.082^b^ ↓ | 1.0 ± 0.072^a^ | 0.64 ± 0.080^b^ ↓ | 1.22 ± 0.094^a^ |
|  | **Atg6** | 1.0 ± 0.052^a^ | 0.82 ± 0.029^a^ | 0.95 ± 0.032^a^ | 1.0 ± 0.069^a^ | 1.15 ± 0.015^a^ | 1.32 ± 0.044^b^ ↑ |
|  | **Atg7** | 1.0 ± 0.026^a^ | 0.77 ± 0.103^a^ | 0.44 ± 0.116^b^ ↓ | 1.0 ± 0.056^a^ | 0.75 ± 0.079^b^ | 0.80 ± 0.109^a^ |
|  | **Atg8a** | 1.0 ± 0.062^a^ | 0.83 ± 0.057^ab^ | 0.71 ± 0.047^b^ ↓ | 1.0 ± 0.072^a^ | 0.52 ± 0.084^b^ ↓ | 1.03 ± 0.041^a^ |
|  | **Atg8b** | 1.0 ± 0.046^a^ | 0.64 ± 0.196^a^ | 1.15 ± 0.088^a^ | 1.0 ± 0.113^a^ | 0.99 ± 0.103^a^ | 1.03 ± 0.103^a^ |
|  | **Atg9** | 1.0 ± 0.014^a^ | 0.99 ± 0.037^a^ | 0.89 ± 0.028^a^ | 1.0 ± 0.032^a^ | 0.86 ± 0.084^a^ | 1.54 ± 0.064^b^ ↑ |
|  | **Atg12** | 1.0 ± 0.055^a^ | 1.26 ± 0.075^b^ ↑ | 0.62 ± 0.057^c^ ↓ | 1.0 ± 0.063^a^ | 1.59 ± 0.067^b^ ↑ | 1.18 ± 0.043^a^ |
|  | **Atg13** | 1.0 ± 0.034^a^ | 1.29 ± 0.137^b^ ↑ | 0.47 ± 0.047^c^ ↓ | 1.0 ± 0.067^a^ | 0.43 ± 0.043^b^ ↓ | 0.67 ± 0.157^b^ ↓ |
|  | **Atg18a** | 1.0 ± 0.043^a^ | 0.86 ± 0.019^a^ | 0.55 ± 0.069^b^ | 1.0 ± 0.084^a^ | 0.88 ± 0.009^a^ | 1.56 ± 0.036^b^ ↑ |
| **Tor** | **AKT** | 1.0 ± 0.022^a^ | 2.09 ± 0.222^b^ ↑ | 1.02 ± 0.080^a^ | 1.0 ± 0.054^a^ | 0.66 ± 0.099^a^ | 0.76 ± 0.092^a^ |
|  | **S6K** | 1.0 ± 0.038^a^ | 1.12 ± 0.127^a^ | 0.93 ± 0.095^a^ | 1.0 ± 0.074^a^ | 0.92 ± 0.022^a^ | 1.32 ± 0.087^b^ ↑ |
|  | **PI3K** | 1.0 ± 0.178^a^ | 1.34 ± 0.168^a^ | 0.50 ± 0.069^b^ ↓ | 1.0 ± 0.014^a^ | 1.03 ± 0.028^a^ | 0.93 ± 0.047^a^ |
|  | **Tor** | 1.0 ± 0.168^a^ | 1.36 ± 0.279^a^ | 1.06 ± 0.152^a^ | 1.0 ± 0.269^a^ | 0.75 ± 0.098^a^ | 0.93 ± 0.474 ^a^ |
|  | **foxo** | 1.0 ± 0.025^a^ | 0.89 ± 0.195^a^ | 0.89 ± 0.167^a^ | 1.0 ± 0.097^a^ | 0.17 ± 0.087^b^ ↓ | 0.50 ± 0.026^c^ ↓ |
| **Gustatory receptors** | **Gr64a** | 1.0 ± 0.154^a^ | 0.53 ± 0.201^a^ | 0.93 ± 0.273^a^ | 1.0 ± 0.137^a^ | 2.49 ± 0.154^b^ ↑ | 1.52 ± 0.138^c^ ↑ |
|  | **Gr64b** | 1.0 ± 0.099^a^ | 1.59 ± 0.197^a^ | 2.39 ± 0.518^a^ | 1.0 ± 0.144^a^ | 1.79 ± 0.214^a^ | 6.97 ± 1.684^b^↑ |
|  | **Gr64c** | 1.0 ± 0.090^a^ | 1.97 ± 0.492^a^ | 1.22 ± 0.596^a^ | 1.0 ± 0.335^a^ | 2.20 ± 1.145^a^ | 5.90 ± 1.426^b^ ↑ |
|  | **Gr64d** | 1.0 ± 0.082^a^ | 2.78 ± 0.232^b^ ↑ | 1.23 ± 0.371^a^ | 1.0 ± 0.095^a^ | 0.65 ± 0.042^b^ ↓ | 0.69 ± 0.071^b^ ↓ |
|  | **Gr64e** | 1.0 ± 0.046^a^ | 2.03 ± 0.208^b^ ↑ | 2.40 ± 0.437^b^ ↑ | 1.0 ± 0.133^a^ | 1.53 ± 0.632^a^ | 2.45 ± 0.244^c^ ↑ |
|  | **Gr64f** | 1.0 ± 0.108^a^ | 4.52 ± 0.505^b^ ↑ | 1.78 ± 0.229^a^ | 1.0 ± 0.142^a^ | 0.47 ± 0.074^b^↓ | 1.22 ± 0.160^a^ |

* ND: not detected. Different letters above the error bars indicate significant differences by one-way ANOVA and Tukey’s HSD post hoc analysis. Significant differences at *p* < 0.05 indicated by ↑ (increase) or ↓ (decrease) compared to those of Nf.

Supplementary Table 3. Differentially expressed genes in AKT/PI3K/Tor-, UPR-, and autophagy signaling and 6 Grs in the head and body of 15-day-old Nf-, GSf-, or GSDf-reared *Drosophila*

| **Genes** | | **Head** | | | **Body** | | |
| --- | --- | --- | --- | --- | --- | --- | --- |
|  |  | **Nf** | **GSf** | **GSDf** | **Nf** | **GSf** | **GSDf** |
| **UPR** | **Hsc70-3** | 1.0 ± 0.104^a^ | 0.71 ± 0.048^b^ ↓ | 0.68 ± 0.035^b^ ↓ | 1.0 ± 0.113^a^ | 0.48 ± 0.067^b^ ↓ | 0.69 ± 0.044^b^ ↓ |
|  | **Ire1** | 1.0 ± 0.062^a^ | 1.20 ± 0.071^a^ | 1.66 ± 0.060^b^ ↑ | 1.0 ± 0.057^a^ | 0.94 ± 0.197^a^ | 1.38 ± 0.045^a^ |
|  | **PEK** | 1.0 ± 0.025^a^ | 1.15 ± 0.124^a^ | 1.74 ± 0.096^b^ ↑ | 1.0 ± 0.110^a^ | 1.52 ± 0.142^b^ ↑ | 0.63 ± 0.070^b^ ↓ |
|  | **ATF6** | 1.0 ± 0.112^a^ | 1.05 ± 0.156^a^ | 1.98 ± 0.008^b^ ↑ | 1.0 ± 0.016^a^ | 1.24 ± 0.109^b^ ↑ | 1.34 ± 0.059^a^ ↑ |
|  | **Xbp1^+23bp^** | 1.0 ± 0.153^a^ | 0.84 ± 0.104^a^ | 0.92 ± 0.026^a^ | 1.0 ± 0.120^a^ | 0.35 ± 0.063^b^ ↓ | 0.84 ± 0.018^a^ |
|  | **Xbp1^-23bp^** | 1.0 ± 0.226^a^ | 0.32 ± 0.032^b^ ↓ | 0.37 ± 0.031^b^ ↓ | 1.0 ± 0.049^a^ | 0.35 ± 0.031^b^ ↓ | 0.49 ± 0.144^b^ ↓ |
|  | **crc** | 1.0 ± 0.034^a^ | 0.92 ± 0.012^a^ | 1.27 ± 0.067^b^ ↑ | 1.0 ± 0.007^a^ | 0.81 ± 0.013^b^ ↓ | 1.01 ± 0.028^a^ |
|  | **Grp-170** | 1.0 ± 0.024^a^ | 0.76 ± 0.048^b^ ↓ | 0.98 ± 0.040^a^ | 1.0 ± 0.077^a^ | 0.87 ± 0.038^a^ | 1.39 ± 0.037^b^ ↑ |
|  | **Gp93** | 1.0 ± 0.089^a^ | 0.57 ± 0.024^b^ ↓ | 0.61 ± 0.005^b^ ↓ | 1.0 ± 0.091^a^ | 0.79 ± 0.044^b^ ↓ | 1.35 ± 0.036^c^ ↑ |
| **Autophagy** | **Hsc70-4** | 1.0 ± 0.087^a^ | 0.83 ± 0.056^a^ | 0.84 ± 0.018^a^ | 1.0 ± 0.038^a^ | 0.64 ± 0.064^b^ ↓ | 0.68 ± 0.095^b^ ↓ |
|  | **Hsc70-5** | 1.0 ± 0.152^a^ | 1.0 ± 0.097^a^ | 1.46 ± 0.039^b^ ↑ | 1.0 ± 0.079^a^ | 0.73 ± 0.054^b^ ↓ | 0.93 ± 0.071^a^ |
|  | **Atg1** | 1.0 ± 0.080^a^ | 1.07 ± 0.101^a^ | 2.03 ± 0.062^b^ ↑ | 1.0 ± 0.050^a^ | 0.44 ± 0.078^b^ ↓ | 1.23 ± 0.024^c^ ↑ |
|  | **Atg2** | 1.0 ± 0.063^a^ | 1.04 ± 0.143^a^ | 1.54 ± 0.013^b^ ↑ | 1.0 ± 0.060^a^ | 1.60 ± 0.022^b^ ↑ | 1.14 ± 0.081^a^ |
|  | **Atg3** | 1.0 ± 0.058^a^ | 1.94 ± 0.142^b^ ↑ | 1.56 ± 0.019^c^ ↑ | 1.0 ± 0.012^a^ | 1.24 ± 0.051^b^ ↑ | 1.27 ± 0.010^b^ ↑ |
|  | **Atg4a** | 1.0 ± 0.082^a^ | 0.62 ± 0.049^b^ ↓ | 1.10 ± 0.069^a^ | 1.0 ± 0.142^a^ | 0.55 ± 0.050^b^ ↓ | 0.64 ± 0.033^a^ ↓ |
|  | **Atg5** | 1.0 ± 0.012^a^ | 1.17 ± 0.095^a^ | 1.46 ± 0.058^b^ ↑ | 1.0 ± 0.018^a^ | 1.18 ± 0.018^b^ ↑ | 1.26 ± 0.065^b^ ↑ |
|  | **Atg6** | 1.0 ± 0.046^a^ | 1.34 ± 0.065^b^ ↑ | 1.35 ± 0.101^b^ ↑ | 1.0 ± 0.029^a^ | 1.27 ± 0.105^a^ | 0.94 ± 0.113^a^ |
|  | **Atg7** | 1.0 ± 0.009^a^ | 1.26 ± 0.131^b^ ↑ | 1.36 ± 0.042^b^ ↑ | 1.0 ± 0.025^a^ | 1.21 ± 0.142^ab^ | 1.38 ± 0.082^b^ ↑ |
|  | **Atg8a** | 1.0 ± 0.046^a^ | 0.92 ± 0.035^a^ | 1.16 ± 0.020^b^ ↑ | 1.0 ± 0.033^a^ | 1.46 ± 0.048^b^ ↑ | 1.02 ± 0.105^a^ |
|  | **Atg8b** | 1.0 ± 0.078^a^ | 1.84 ± 0.267^b^ ↑ | 2.01 ± 0.063^b^ ↑ | 1.0 ± 0.032^a^ | 1.47 ± 0.085^b^ ↑ | 1.73 ± 0.018^c^ ↑ |
|  | **Atg9** | 1.0 ± 0.028^a^ | 0.98 ± 0.085^a^ | 1.05 ± 0.035^a^ | 1.0 ± 0.002^a^ | 1.14 ± 0.086^a^ | 1.20 ± 0.096^a^ |
|  | **Atg12** | 1.0 ± 0.041^a^ | 1.43 ± 0.030^b^ | 1.25 ± 0.044^c^ | 1.0 ± 0.099^a^ | 1.30 ± 0.071^b^ ↑ | 1.39 ± 0.072^b^ ↑ |
|  | **Atg13** | 1.0 ± 0.042^a^ | 0.98 ± 0.157^a^ | 1.60 ± 0.030^b^ | 1.0 ± 0.008^a^ | 0.94 ± 0.106^a^ | 1.06 ± 0.092^a^ |
|  | **Atg18a** | 1.0 ± 0.281^a^ | 0.25 ± 0.022^b^ ↓ | 0.07 ± 0.046^b^ ↓ | 1.0 ± 0.053^a^ | 0.98 ± 0.060^a^ | 1.11 ± 0.047^a^ |
| **Tor** | **AKT** | 1.0 ± 0.052^a^ | 0.89 ± 0.061^a^ ↑ | 1.06 ± 0.041^a^ ↑ | 1.0 ± 0.088^a^ | 1.12 ± 0.117^a^ | 0.98 ± 0.166^a^ |
|  | **S6K** | 1.0 ± 0.131^a^ | 1.50 ± 0.171^b^ ↑ | 2.0 ± 0.126^c^ ↑ | 1.0 ± 0.042^a^ | 1.44 ± 0.087^b^ ↑ | 1.15 ± 0.106^a^ |
|  | **PI3K** | 1.0 ± 0.092^a^ | 0.87 ± 0.137^a^ | 0.91 ± 0.094^a^ | 1.0 ± 0.083^a^ | 0.90 ± 0.041^a^ | 0.77 ± 0.028^b^ ↓ |
|  | **Tor** | 1.0 ± 0.232^a^ | 0.77 ± 0.175^a^ | 0.56 ± 0.022^a^ | 1.0 ± 0.041^a^ | 0.82 ± 0.081^b^ ↓ | 0.44 ± 0.029 ^c^ ↓ |
|  | **foxo** | 1.0 ± 0.065^a^ | 1.02 ± 0.061^a^ | 1.09 ± 0.015^a^ | 1.0 ± 0.045^a^ | 1.09 ± 0.022^a^ | 0.82 ± 0.055^b^ ↓ |
| **Gustatory receptors** | **Gr64a** | 1.0 ± 0.247^a^ | 1.70 ± 0.086^b^ ↑ | 2.29 ± 0.146^c^ ↑ | 1.0 ± 0.085^a^ | 1.72 ± 0.547^a^ | 1.55 ± 0.213^a^ |
|  | **Gr64b** | 1.0 ± 0.089^a^ | 2.94 ± 0.193^b^ ↑ | 1.22 ± 0.291^a^ | 1.0 ± 0.223^a^ | 0.12 ± 0.044^b^ ↓ | 0.64 ± 0.019^a^ |
|  | **Gr64c** | 1.0 ± 0.151^a^ | 2.38 ± 0.147^b^ ↑ | 1.91 ± 0.129^c^ ↑ | 1.0 ± 0.212^a^ | 0.28 ± 0.120^b^ ↓ | 0.27 ± 0.082^b^ ↓ |
|  | **Gr64d** | 1.0 ± 0.082^a^ | 0.70 ± 0.148^a^ | 0.87 ± 0.045^a^ | 1.0 ± 0.090^a^ | 0.47 ± 0.075^b^ ↓ | 0.62 ± 0.054^b^ ↓ |
|  | **Gr64e** | 1.0 ± 0.046^a^ | 1.0 ± 0.136^a^ | 1.37 ± 0.040^a^ | 1.0 ± 0.090^a^ | 0.98 ± 0.073^a^ | 1.76 ± 0.068^b^ ↑ |
|  | **Gr64f** | 1.0 ± 0.108^a^ | 1.39 ± 0.109^b^ ↑ | 0.60 ± 0.072^c^ ↓ | 1.0 ± 0.155^a^ | 0.80 ± 0.088^a^ | 1.39 ± 0.099^b^ ↑ |

* ND: not detected. Different letters above the error bars indicate significant differences by one-way ANOVA and Tukey’s HSD post hoc analysis. Significant differences at *p* < 0.05 indicated by ↑ (increase) or ↓ (decrease) compared to those of Nf.

**2 Supplementary Figure**

Supplementary Figure 1. Altered biophysical characteristics of GS digested with a food-grade protease revealed by size exclusion chromatography

A. The chromatogram of a RIPA extract of GS had two separate parts. The size of the 1^st^ part was smaller than that of the 2^nd^ part. B. The chromatogram of a RIPA extract of GSD had the tiny 1^st^ part and the large 2^nd^ part. C. The chromatogram of a RIPA extract of GSDsup showed that the narrow and pointed 1^st^ part appeared close to the large 2^nd^ part. D. The chromatogram of a RIPA extract of GSDprec showed two part. The 1^st^ part of the GSDprec-RIPA extract was larger than those of other samples. Blue lines indicated UV absorbance graphs at 280 nm. Red lines indicated UV absorbance graphs at 254 nm. Purple lines indicated UV absorbance graphs at 215 nm.

Supplementary Figure 2. The activities of mitochondria complexes (MitoComs) I ~ IV in Nf-, GSf-, or GSDf-reared *Drosophila*

Normalized activities of MitoCom I (A), II (B), III (C), and IV (D) in Nf-, GSf-, or GSDf-reared *Drosophila* on days 5, 10, 15, and 20. (A) Day 5, F_(2, 15)_ = 23.9005, *p* = 2.2 x 10^-5^; Day 10, F_(2, 15)_ = 20.70075, *p* = 4.85 x 10^-6^; Day 15, F_(2, 15)_ = 186.3258, *p* = 9.36 x 10^-13^; Day 20, F_(2, 15)_ = 7.069456, *p* = 0.0034. (B) Day 5, F_(2, 15)_ = 21.5483, *p* = 3.9 x 10^-5^; Day 10, F_(2, 15)_ = 176.0277, *p* = 3.85 x 10^-11^; Day 15, F_(2, 15)_ = 444.5335, *p* = 4.46 x 10^-14^; Day 20, F_(2, 15)_ = 25.6584, *p* = 1.87 x 10^-7^. (C) Day 5, F_(2, 15)_ = 22.571, *p* = 5.9 x 10^-6^; Day 10, F_(2, 15)_ = 30.92501, *p* = 4.8 x 10^-6^; Day 15, F_(2, 15)_ = 165.6444, *p* = 5.96 x 10^-11^; Day 20, F_(2, 15)_ = 18.15284, *p* = 2.64 x 10^-5^. (D) Day 5, F_(2, 15)_ = 6.41272, *p* = 0.00971; Day 10, F_(2, 15)_ = 59.84265, *p* = 7.09 x 10^-8^; Day 15, F_(2, 15)_ = 176.7715, *p* = 3.73 x 10^-11^; and Day 20, F_(2, 15)_ = 135.7819, *p* = 8.14 x 10^-15^. The letters above the error bars represent significant differences as determined by one-way ANOVA and Tukey’s HSD *post hoc* test.


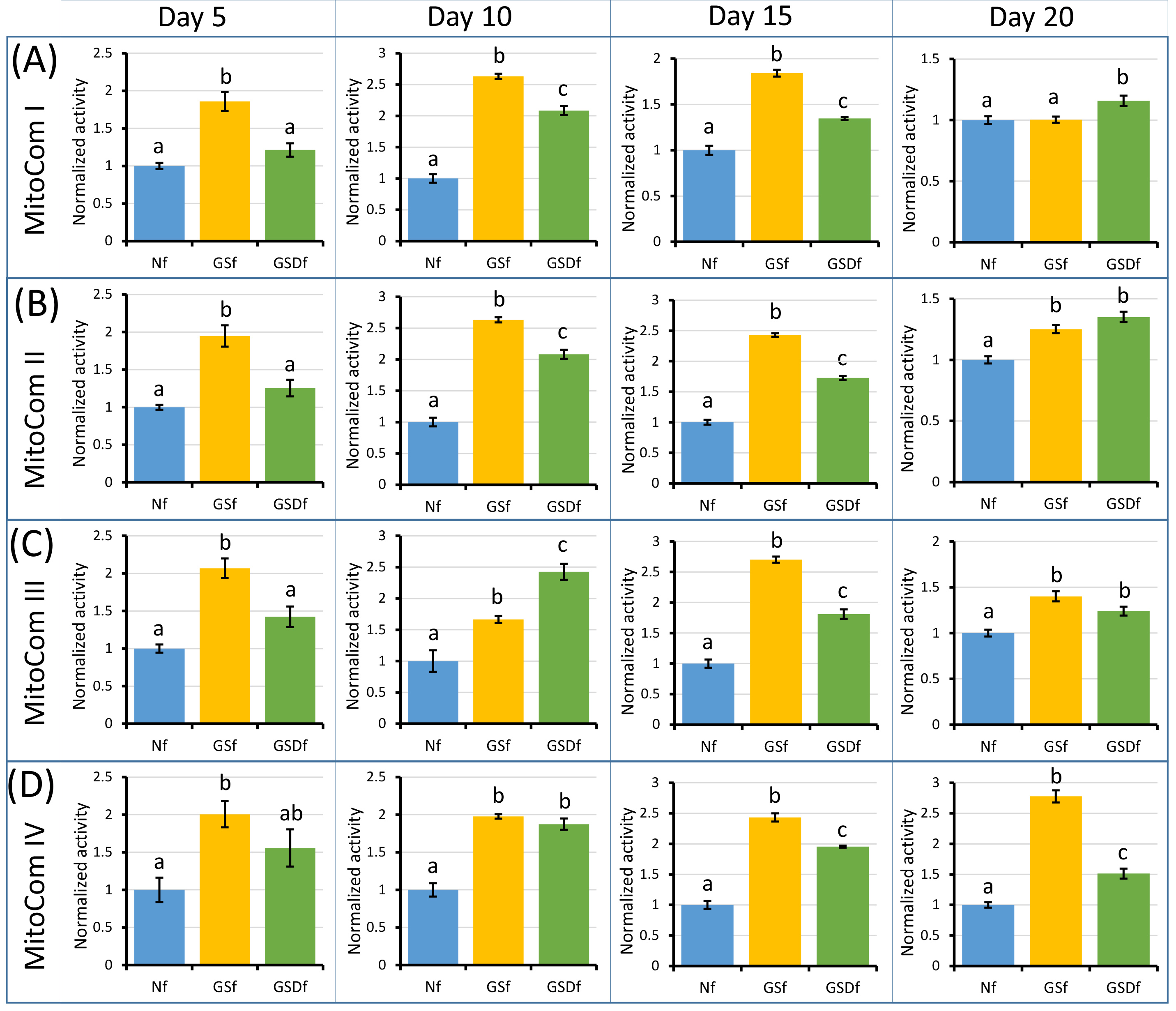

Supplement: Supplementary file 1 [file Table_1.DOCX]
